# Anti‐tuberculosis effect of isoniazid scales accurately from zebrafish to humans

**DOI:** 10.1111/bph.15247

**Published:** 2020-11-03

**Authors:** Rob C. van Wijk, Wanbin Hu, Sharka M. Dijkema, Dirk‐Jan van den Berg, Jeremy Liu, Rida Bahi, Fons J. Verbeek, Ulrika S.H. Simonsson, Herman P. Spaink, Piet H. van der Graaf, Elke H.J. Krekels

**Affiliations:** ^1^ Division of Systems Biomedicine and Pharmacology, Leiden Academic Centre for Drug Research Leiden University Leiden The Netherlands; ^2^ Division of Animal Sciences and Health, Institute of Biology Leiden Leiden University Leiden The Netherlands; ^3^ Imaging and Bioinformatics Group, Leiden Institute of Advanced Computer Science Leiden University Leiden The Netherlands; ^4^ Department of Pharmaceutical Biosciences Uppsala University Uppsala Sweden; ^5^ QSP Certara Canterbury UK

**Keywords:** Imaging, in vivo, mathematical modelling, pharamacodynamics, pharmacokinetics, Translational pharmacology, tuberculosis, zebrafish

## Abstract

**Background and Purpose:**

There is a clear need for innovation in anti‐tuberculosis drug development. The zebrafish larva is an attractive disease model in tuberculosis research. To translate pharmacological findings to higher vertebrates, including humans, the internal exposure of drugs needs to be quantified and linked to observed response.

**Experimental Approach:**

In zebrafish studies, drugs are usually dissolved in the external water, posing a challenge to quantify internal exposure. We developed experimental methods to quantify internal exposure, including nanoscale blood sampling, and to quantify the bacterial burden, using automated fluorescence imaging analysis, with isoniazid as the test compound. We used pharmacokinetic–pharmacodynamic modelling to quantify the exposure–response relationship responsible for the antibiotic response. To translate isoniazid response to humans, quantitative exposure–response relationships in zebrafish were linked to simulated concentration–time profiles in humans, and two quantitative translational factors on sensitivity to isoniazid and stage of infection were included.

**Key Results:**

Blood concentration was only 20% of the external drug concentration. The bacterial burden increased exponentially, and an isoniazid dose corresponding to 15 mg·L^−1^ internal concentration (minimum inhibitory concentration) leads to bacteriostasis of the mycobacterial infection in the zebrafish. The concentration–effect relationship was quantified, and based on that relationship and the translational factors, the isoniazid response was translated to humans, which correlated well with observed data.

**Conclusions and Implications:**

This proof of concept study confirmed the potential of zebrafish larvae as tuberculosis disease models in translational pharmacology and contributes to innovative anti‐tuberculosis drug development, which is very clearly needed.

**Abbreviations**
ADC: acid–albumin–dextrose–catalase
CFU: colony‐forming units
dpf: days post fertilization
dpi: days post infection
FOCE: first‐order conditional estimation
GI: gastrointestinal
hpf: hours post fertilization
LLOQ: lower limit of quantification
MGIT: mycobacteria growth indicator tube
MIC: minimum inhibitory concentration
MTP: multistate tuberculosis pharmacometric
OADC: oleic acid–albumin–dextrose–catalase
PVP40: polyvinylpyrrolidone 40
QC: quality control
TB: tuberculosis

**What is already known**

- The zebrafish is a well‐known tuberculosis disease model for qualitative efficacy screens in drug development.
- The information content and translational potential of qualitative assessments are low.

**What this study adds**

- Isoniazid exposure–response relationship was quantified in zebrafish larvae using integrated experimental and computational innovation.
- With pharmacokinetic–pharmacodynamic modelling, quantitative translation of pharmacological findings from zebrafish larvae to humans is confirmed.

**What is the clinical significance**

- Adding zebrafish larvae to the translational drug development pipeline could expedite development of anti‐tuberculosis drugs.

## 1 INTRODUCTION

Tuberculosis (TB) is the leading cause of death from infectious diseases in adults, and *Mycobacterium tuberculosis* is becoming the deadliest pathogen on the planet (Furin, Cox, & Pai, 2019). The United Nations Sustainable Development Goals aim to eradicate the TB epidemic before 2030, but progress is stalling due to ineffectiveness of currently available treatments (United Nations, 2019). Drug development is a challenging, lengthy and costly process, generally requiring a decade for drugs to reach the market with estimated costs of $1–2.5 billion per approved new drug (DiMasi, Grabowski, & Hansen, 2016). Development of anti‐TB drugs is especially difficult, because of the laboratory biosafety issues (World Health Organization, 2012), the slow replication rate of *M. tuberculosis*, and the long duration of treatment and patient follow‐up (Ginsberg & Spigelman, 2007). As a result, there is a clear and urgent need for innovations in the development of new TB treatments (Ginsberg & Spigelman, 2007; Van Wijk, Ayoun Alsoud, Lennernäs, & Simonsson, 2020).

Phenotypic‐ and systems‐based drug development both integrate experimental and computational innovation and utilize whole‐organism studies, preferably in vertebrates, for quantitative translational purposes to open a new realm of possible discoveries that are easily overlooked when focusing on single cell‐type targets. High‐throughput experiments within whole vertebrates may improve efficiency and effectiveness of drug development and are possible with the zebrafish larva as model organism (Schulthess et al., 2018).

The zebrafish (*Danio rerio*) is increasingly used in biomedical research, because of its many advantages, which include high fecundity, rapid development, transparency throughout the first period of life, easy genetic modification, and limited ethical constraints (Rennekamp & Peterson, 2015; Schulthess et al., 2018; Van Wijk, Krekels, Hankemeier, Spaink, & Van der Graaf, 2016). Zebrafish larvae infected by *Mycobacterium marinum*, a close relative of *M. tuberculosis*, are an established TB disease model to study host–pathogen interaction (Meijer, 2016; Meijer & Spaink, 2011) and to screen for novel drugs (Carvalho et al., 2011; Ordas et al., 2015), with faster replication times and less biosafety risks than experiments with *M. tuberculosis* (Tobin & Ramakrishnan, 2008). Experiments in zebrafish larvae are commonly performed before the moment they start independent feeding (Rennekamp & Peterson, 2015), which is ethically preferable (Strähle et al., 2012). Other important pathophysiological aspects of TB such as granuloma formation and the drug effect thereon can be studied in later stages of zebrafish development (Van Wijk, Ayoun Alsoud, et al., 2020), but these aspects will not be considered further in this paper.

Imaging of zebrafish larvae infected with fluorescent mycobacteria (Stoop et al., 2011) allows for repeated longitudinal measurements from individual larvae, which reduces overall noise in data as biological and experimental variability can be quantified separately. This is in contrast to ex vivo bacterial burden organ count by colony‐forming units (CFU) or mycobacteria growth indicator tube (MGIT) liquid media systems currently utilized in preclinical TB research (Kolibab, Yang, Parra, Derrick, & Morris, 2014; Kumar et al., 2014).

Translation of drug response between species is challenging and considerably limits drug development (Bartelink et al., 2017). Currently, pharmacological treatment of zebrafish larvae is performed by dissolving drugs into the water in which the larvae swim, without taking into account how much drug is actually taken up by the larvae. Translating pharmacological response between species however requires quantification of the drug exposure at the site of action as a basis for the quantification of the exposure–response relationship (Morgan et al., 2012; Van Wijk, Ayoun Alsoud, et al., 2020). Although such quantification is a challenge because of the small size of the larvae, we have developed new experimental methods to quantify internal drug exposure based on ultra‐sensitive analytical techniques and a novel method for nanoscale blood sampling. Pharmacological model‐based approaches can then be used to quantitatively link the internal exposure over time (pharmacokinetics) of anti‐TB drugs to bactericidal response in the zebrafish larvae observed by fluorescence microscopy (pharmacodynamics). The exposure–response relationship that is thus obtained is the basis for translational pharmacology to higher vertebrates, including humans.

**FIGURE 1:**
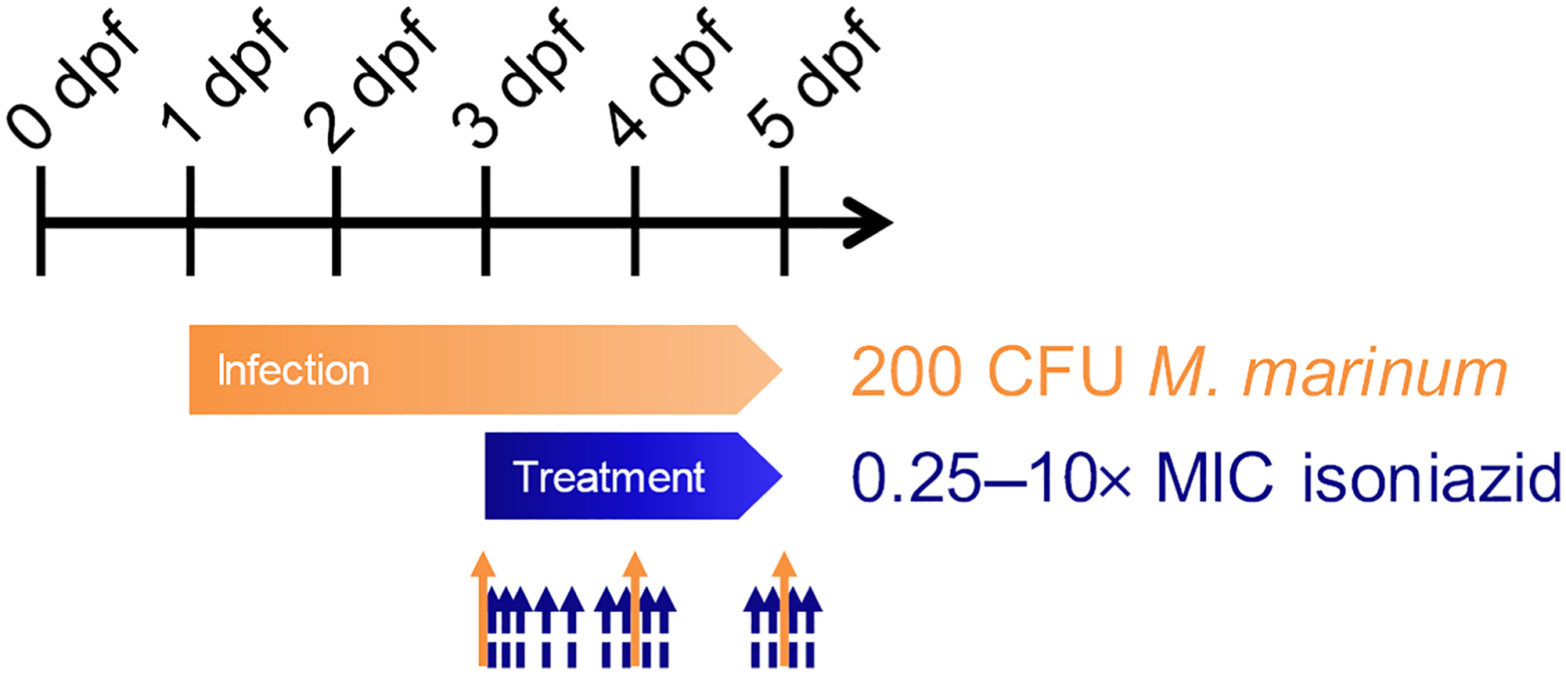
Experimental study design. Fertilized eggs at 0 days post fertilization (dpf) were harvested and injected with 200 CFU *Mycobacterium marinum* at 1 dpf. After 2 days of establishing the infection, fluorescence imaging was performed (orange arrow; *n* ≥ 20 larvae per group), and the treatment with isoniazid dissolved in the external treatment medium (0.25–10‐fold MIC, MIC = 15 mg·L^−1^, and control) was started. Fluorescence imaging was repeated daily (orange solid arrows). In satellite larvae groups, destructive homogenate and blood samples for internal isoniazid exposure quantification were taken from 0 to 50 h of treatment (blue dashed arrows)

Here, for the first time, we present an integration of experimental and computational approaches in preclinical TB research, using zebrafish larvae infected with *M. marinum* and treated with increasing doses of waterborne isoniazid, from 0.25 to 10 times the minimum inhibitor concentration (MIC) (3.75–150 mg·L^−1^). The internal exposure is quantified in homogenates and blood samples of the larvae, and the bacterial burden is quantified by automated fluorescence image analysis, according to the design in Figure 1. Pharmacokinetic–pharmacodynamic modelling was performed to quantify the exposure over time and the exposure–response relationship. Isoniazid was chosen because it is known to have the largest early bactericidal activity for single drug treatments among the current standard of care drugs against TB (Jindani, Aber, Edwards, & Mitchison, 1980). The quantified exposure–response relationship in the zebrafish larvae together with simulated concentration–time profiles in TB patients was utilized to translate the findings on isoniazid response in the zebrafish larvae to humans. A quantitative comparison with reported observations from patients was made as a proof of concept, to assess translational value of this new disease model in anti‐TB drug development.

## 2 METHODS

### 2.1 Study design

Zebrafish embryos of the VUmc wild‐type strain were dechorionated and infected with *M. marinum* strain E11 at 28 hours post fertilization (hpf) and kept at 28°C throughout the experiment. At 2 days post infection (dpi), the bacterial burden in zebrafish larvae was quantified by fluorescence microscopy, and waterborne treatment with isoniazid was commenced at external concentrations of 0, 3.75, 7.5, 15, 30, and 75 mg·L^−1^ corresponding to 0, 0.25, 0.5, 1, 2, and 5× MIC. Quantification of the bacterial burden was repeated within individual larva at 3 and 4 dpi to assess individual early bactericidal response. Internal isoniazid exposure was quantified by LC–MS/MS of whole zebrafish larval homogenate samples as well as in larval blood samples in a parallel experiment with uninfected larvae treated with external isoniazid concentrations of 7.5, 15, 30, 75, and 150 mg·L^−1^ corresponding to 0.5, 1, 2, 5, and 10× MIC. All replicates are biological replicates; technical replicates were not obtained. Figure 1 shows a schematic overview of the study design.

### 2.2 Zebrafish husbandry

Zebrafish were maintained and handled following international consensus protocols (Westerfield, 2000). Planning and execution of all experiments complied with European regulation, in which zebrafish embryos and larvae are not considered experimental animals throughout the duration of our experiments (0–5 days post fertilization [dpf]) (European Union [EU], 2010). Adult wild‐type VUmc zebrafish were kept in glass aquaria (max 6 L^−1^, volume 10 L, 120 × 220 × 490 mm; Fleuren & Nooijen BV, Nederweert, The Netherlands) with circulating water (27.7°C ± 0.1 on a 14 h/10 h light/dark cycle, lights on at 08:00). Adult zebrafish were fed twice daily with artemia or feed particles (Gemma Micro/Diamond, Skretting, Nutreco NV, Amersfoort, The Netherlands). JUMO ACQUIS touch S (JUMO GmbH & Co., Weesp, The Netherlands) was used to control water quality.

Adult zebrafish were set‐up for breeding overnight, and in the morning at lights on, separators between males and females were removed, and fertilized eggs were collected within 30 min of fertilization. Eggs, embryos, and larvae were kept in embryo medium (60 μg·ml^−1^ Instant Ocean Sea Salts [Sera, Heinsberg, Germany] in demineralized water, daily refreshed) at 28°C. For the duration of treatment, larvae were kept in embryo medium with isoniazid at 28°C until imaging or sampling.

### 2.3 Internal exposure of isoniazid in zebrafish larvae

Internal exposure of isoniazid in zebrafish larvae was quantified as drug amounts in larval homogenates or as drug concentrations in blood samples after waterborne treatment with isoniazid concentrations at external concentrations of 0.5, 1, 2, 5, and 10× MIC (7.5, 15, 30, 75, and 150 mg·L^−1^). Experiments were performed on different days per dose, and the experimentalist was unblinded to the external concentration when blood samples or homogenates were obtained. As no outcome measurements were acquired at this stage, this was not expected to result in measurement bias.

Homogenate samples of pooled samples with five larvae at time points 0.25, 0.5, 1, 2, 3, 6, 8, 9, 18, 20, 22, 24, 26, 32, 42, 44, 46, 48, and 50 h after start of treatment were obtained at *n* = 3, except for external concentration of 1× MIC (15 mg·L^−1^), which was repeated to check for inter‐day variability, thus resulting in *n* = 6 replicates. Zebrafish larvae were washed 4 times with 20/80 methanol/water (v/v) using Netwell inserts (Corning Life Sciences B.V., Amsterdam, The Netherlands) and transferred to Safe‐Lock tubes (Eppendorf Nederland B.V., Nijmegen, The Netherlands). Excess volume was removed, and 50 μl of 200 ng·ml^−1^ isoniazid‐D4 internal standard was added after which the larvae were snap frozen in liquid nitrogen and stored at −80°C until quantification.

Blood samples (*n* = 4) were taken after 48 h of treatment at the highest external concentration of 150 mg·L^−1^ to ascertain quantifiable levels, using a previously published method (Van Wijk, Krekels, Kantae, Ordas, et al., 2019). In short, zebrafish larvae of 5 dpf were washed 4 times as described above while being unanaesthetized to prevent bradycardia and decreased sample yield. The larvae were superficially dried and transferred to an agarose microscopy slide, after which a pulled needle (0.75 mm borosilicate class capillary without filament; Sutter Instruments, Novato, California, USA) positioned in a micromanipulator (World Precision Instruments, Berlin, Germany) attached to a CellTram Vario oil pump (Eppendorf Nederland B.V., Nijmegen, The Netherlands) was used to sample the blood from the posterior cardinal vein under 20× magnification (Leica, Amsterdam, The Netherlands). Blood was sampled until the larva was exsanguinated. An image of each blood sample was captured to calculate the obtained blood volume before the sample was injected into a 2‐μl heparin droplet (5 IU·ml^−1^) and pooled into a 0.5‐ml tube (Eppendorf Nederland B.V.). Blood samples were kept at −80°C until quantification. Table S1 reports the number of larvae sampled per pooled sample, as well as the blood volume and isoniazid concentration per time point.

### 2.4 Quantification of isoniazid in homogenate and blood samples

Isoniazid was quantified by LC–MS/MS. Derivatization with cinnamaldehyde was performed to achieve adequate retention for LC separation. The operator was unblinded, and to reduce measurement bias, all samples were randomized prior to injection into the LC–MS/MS, and peak processing was automated (LCQuan software v. 2.7, Thermo Fisher Scientific, Breda, The Netherlands).

Samples of whole zebrafish larva were thawed, and 100 μl methanol and 100 μl of 0.5 mm zirconium oxide bullets (NextAdvance, New York, USA) were added. The samples were homogenized using a Bullet Blender (NextAdvance) for at least two rounds of 5 min at speed 5. Extraction and derivatization were performed by adding 800 μl acetonitrile, 100 μl 1% cinnamaldehyde in methanol, and 100 μl formic acid and shaking for 20 min at 650 r.p.m. (IKA, Staufen im Breisgau, Germany). Samples were centrifuged for 10 min at 20,000× *g*; 90% was transferred to a new Safe‐Lock tube and evaporated until dryness in a Labconco vacuum centrifuge (Beun de Ronde, Abcoude, The Netherlands). The residue was reconstituted into 200 μl methanol, centrifuged for 10 min at 20,000× *g*, and the supernatant was transferred to an LC–MS/MS vial with a glass insert for 5 μl injection into the LC–MS/MS. Quality control (QC) and calibration curve samples were prepared by adding known amounts to blank homogenate samples. The QC samples were prepared at the levels of 4, 125, and 225 ng·ml^−1^. The calibration curve samples were prepared at the levels of 0, 2, 5, 10, 25, 50, 100, 150, and 250 ng·ml^−1^.

Blood samples were thawed and briefly centrifuged to concentrate the small sample into the bottom of the tube. A 2.5 μl of 200 ng·ml^−1^ internal standard solution was added. Extraction and derivatization were performed by adding 200 μl acetonitrile, 50 μl methanol, 25 μl 1% cinnamaldehyde in methanol, and 25 μl formic acid, and samples were shaken for 20 min at 650 r.p.m. Samples were evaporated until dry, reconstituted in 10 μl methanol, and centrifuged for 10 min at 20,000× *g*, after which they were transferred to an LC–MS/MS vial with a glass insert upon 5 μl injection into the LC–MS/MS. An academic calibration curve, in methanol without biological matrix, was prepared with concentrations 0, 2, 3, 5, 8, 10, 25, 50, 100, 150, and 250 ng·ml^−1^.

Quantification of isoniazid was performed on an ultra HPLC system (Shimadzu Nexera X2, 's‐Hertogenbosch, The Netherlands) with a triple quadrupole MS detector (TSQ Vantage, Thermo Fisher Scientific). Electron spray ionization in positive modes was used to obtain derivative ions.

Chromatography was performed at a flow of 0.4 ml·min^−1^ on a Luna Omega Polar C18 1.7 μm 100 × 2.1 mm column with a 5‐mm guard column with the same packing material (Phenomenex, Utrecht, The Netherlands). The temperature of the column was maintained at 40°C. Gradient elution was performed with two ultra HPLC pumps using methanol/water mixtures with 0.01% formic acid. The gradient started at the time of injection and increased from a 49/51 methanol/water v/v ratio to a 72/28 methanol/water v/v ratio within 4 min. The column was flushed with 95/5 methanol/water v/v ratio starting at 4.1 min for 2.4 min, after which the system was equilibrated to initial conditions.

Within the MS system, the vaporizer temperature was set at 300°C and capillary temperature at 250°C. Sheath gas pressure was 40 psi, and multiple reaction monitoring was used to quantify the isoniazid derivative (MH^+^ = 252.1 m/z) and the isoniazid‐D4‐adduct (MH^+^ = 256.1 m/z). For the isoniazid derivative, the fragments were 79.01 and 121.01 m/z, and for the internal standard derivative, the fragments were 83.10 and 124.96 m/z. The sheath gas pressure was 40 psi, auxiliary gas pressure was 15 psi, S‐lens RF amplitude was 63 V, and capillary pressure was 1.190 mTorr. Detection limit was 0.5 ng·ml^−1^, and lower limit of quantification (LLOQ) was 1.75 ng·ml^−1^. The LC–MS/MS method was validated according to the US Food and Drug Administration guidelines (US Food and Drug Administration, 2018). LCQuan software was used for data acquisition where isoniazid peak area was corrected by internal standard peak area, and calibration was performed with weighted linear regression using 1/y as weighting factor. Of the pharmacokinetic data points, 3% were below the LLOQ, and 1% were above the highest calibration standard; these samples were excluded from the analysis (Beal, 2001).

### 2.5 Bacterial strain preparation

The bacterial strain *M. marinum* E11 expressing mCherry fluorescent protein (Van der Sar, Spaink, Zakrzewska, Bitter, & Meijer, 2009) was used to induce an infection in zebrafish embryos. *Mycobacterium marinum* E11 was cultured and harvested as previously described (Benard et al., 2012). In short, a colony of *M. marinum* E11 was picked from 7H10 supplemented with 10% oleic acid‐albumin‐dextrose‐catalase (OADC), and the colony was suspended in 7H9 supplemented with 10% acid‐albumin‐dextrose‐catalase (ADC) and cultured overnight at 28°C. The OD at 600 nm (OD_600_) of bacteria was measured the next day (Eppendorf Biophotometer 6131, Eppendorf, Hamburg, Germany). The logarithmic phase bacteria were harvested and washed 3 times with sterile PBS. The infection inoculum was resuspended to a target concentration of 200 CFU·nl^−1^ in 2% polyvinylpyrrolidone‐40 solution (PVP40). The MIC of the used strain had been determined to be 15 mg·L^−1^, in line with reported values (Aubry, Jarlier, Escolano, Truffot‐Pernot, & Cambau, 2000).

### 2.6 *Mycobacterium marinum* bacterial burden in zebrafish larvae upon isoniazid treatment

The injection of zebrafish larvae with *M. marinum* was performed as described previously (Benard et al., 2012). Briefly, at 24 hpf, zebrafish embryos were dechorionated manually with fine tweezers (F6521‐1E Jewelers forceps, Dumont No. 5, Sigma‐AldrichChemie GmbH [Schnelldorf, Germany]). The microinjection needles (BF100‐75‐10, Sutter Instruments) were prepared with a micropipette puller device (P‐97 Flaming/Brown Micropipette Puller, Sutter Instrument). Embryos of 28 hpf were anaesthetized with 200 μg·ml^−1^ ethyl 3‐aminobenzoate (tricaine), 10 min prior to injection and injected with 1 nl of 200 CFU·nl^−1^ *M. marinum* E11 using the microinjection system (FemtoJet, Eppendorf, Hamburg, Germany), into the caudal vein at the blood island. After injection, the embryos were kept at 28°C. To quantify the established infection at 2 dpi, a Fluorescence Stereo Microscope (Leica MZ16FA, Leica Microsystems, Wetzlar, Germany) equipped with the digital camera (Leica DFC420 C, Leica Microsystems) was utilized for fluorescence imaging, after which the larvae were transferred to 96‐well plates (655180, Greiner Bio‐One, Kremsmünster, Austria) with isoniazid solutions of 0, 3.75, 7.5, 15, 30, or 75 mg·L^−1^ in embryo medium. Group size was designed at *n* = 20, and final group size taking into account experimental loss of larvae was *n* = 17, *n* = 20, *n* = 17, *n* = 19, *n* = 18, and *n* = 19 for 0, 3.75, 7.5, 15, 30, or 75 mg·L^−1^ isoniazid, respectively. At 3 and 4 dpi, fluorescence imaging of individual larvae was repeated, after which they were transferred to a new 9‐well plate with fresh isoniazid treatment solution (3 dpi) or sacrificed by tricaine overdose (4 dpi).

The experimenter was unblinded to the dose, but the potential of measurement bias was limited as image analysis was automated. Automated image analysis was performed as reported before (Nezhinsky & Verbeek, 2010; Stoop et al., 2011), where count of pixels with fluorescence is assumed to correlate directly with the bacterial burden. Possible differences in the bacterial burden between individual larvae at the start of treatment were tested by non‐parametric Kruskal–Wallis test; 12.7% (16/126) of the larvae were removed from the data set due to improper injection, developmental defects (e.g., cardiac oedema), or death due to mechanical damage from handling or otherwise.

### 2.7 Quantification of the exposure–response relationship for isoniazid in zebrafish larvae

Data were analysed by non‐linear mixed effects modelling, which was used to develop a pharmacokinetic–pharmacodynamic model that quantified the internal exposure over time and the exposure–response relationship of isoniazid on bacterial growth, using ordinary differential equations. For this type of analysis, it is not necessary that groups are of equal sizes or that samples are taken at the exact same time for each treatment group. Moreover, this method does not allow for an *a priori* assessment of statistical power or group size calculations. NONMEM (version 7.3, ICON Development Solutions, Ellicott City, MD, USA; RRID:SCR_016986) (Beal, Sheiner, Boeckmann, & Bauer, 2013) through interfaces Pirana (version 2.9.6) (Keizer, Van Benten, Beijnen, Schellens, & Huitema, 2011) and PsN (version 4.7.0) (Lindbom, Pihlgren, & Jonsson, 2005) was used for non‐linear mixed effects modelling. R (version 3.5.0, R. Core Team, Vienna, Austria; RRID:SCR_001905) through the RStudio interface (version 1.1.383, RStudio Inc., Boston, Massachusetts, USA; RRID:SCR_000432) was used for data transformation and graphical output. The first‐order conditional estimation (FOCE) algorithm with interaction was used for pharmacokinetic–pharmacodynamic modelling.

In the pharmacokinetic component of the model, the homogenate and blood sample data were fitted simultaneously. The treatment medium was represented as depot compartment from which a first‐order absorption rate constant into a one‐compartment model with distribution volume and linear or non‐linear (Michaelis–Menten) elimination was estimated. Concentration in the treatment medium was assumed to be constant, as supported by measurements of external drug concentrations (Figure S1).

We expected age to be a covariate (predictor) on the absorption and elimination rate constants, but because the effects of age on absorption and elimination are indistinguishable at steady state, we have estimated a net effect of age as covariate for a net increase on absorption only, which reflects the relative increase (or decrease in case of a negative value) in absorption rate constant, compared to the increase in elimination rate constant. For this, we tested a linear, power, or exponential relationship. In addition, a discrete increase in absorption rate constant between 3 and 4 dpf to reflect the effects of the opening of the gastrointestinal (GI) tract (Van Wijk, Krekels, Kantae, Harms, et al., 2019) was added. Out of additive, proportional, or combination error models, the residual error, describing biological and experimental error, was best described by a combination of an additive and proportional error model for the isoniazid amounts in homogenates and a proportional error model for isoniazid concentrations in the blood samples. A visual predictive check of the pharmacokinetic component of the model was performed using 500 simulations, stratified per dose.

The pharmacodynamic component of the model was fitted through log_10_ transformed data of the bacterial burden. Bacterial growth was tested using exponential (Equation 1), Gompertz (Equation 2), or logistic (Equation 3) growth functions.

(1) $$\frac{\text{dBac}}{\mathrm{dt}}=k_{g}\cdot \mathrm{Bac},$$

(2) $$\frac{\text{dBac}}{\mathrm{dt}}=k_{g}\cdot \log\left(\frac{B_{\max}}{\mathrm{Bac}}\right),$$

(3) $$\frac{\text{dBac}}{\mathrm{dt}}=k_{g}\cdot \log\left(B_{\max}-\mathrm{Bac}\right),$$

in which Bac represented the bacterial burden, *t* time (h), *k*_g_ the growth rate (h^−1^), and *B*_max_ the maximum capacity of the system (log_10_ fluorescence). Because no ceiling of bacterial growth was observed in the data, the maximum capacity of the system could not be estimated for the Gompertz or logistic growth function. Growth and decay could not be estimated separately as this was mathematically not identifiable, given the data. Therefore, a net effect was estimated for growth, which means a negative growth represents a net decay or kill of bacteria.

Based on the homogenate and blood sample data at 5 dpf, the distribution volume at this age was estimated. This distribution volume was subsequently scaled to 3 and 4 dpf based on total larval volume quantified previously (Guo, Veneman, Spaink, & Verbeek, 2017). The pharmacokinetic component of the model converted total isoniazid amounts from homogenates to concentrations using these distribution volumes (Equation 4).

(4) $$C_{\mathrm{INH}}=\frac{A_{\mathrm{INH}}}{V_{d}},$$

in which *C*_INH_ is the concentration of isoniazid (ng·μl^−1^ or mg·L^−1^), *A*_INH_ is the amount of isoniazid (ng) in the homogenate samples, and *V*_d_ is the scaled volume of distribution (μl) on each day.

For the exposure–response relationship (EFF), linear (Equation 5), *E*_max_ (Equation 6), or sigmoidal *E*_max_ (Equation 7) functions were tested.

(5) $$\mathrm{EFF}=\mathrm{SLP}\cdot C_{\mathrm{INH}},$$

(6) $$\mathrm{EFF}=\frac{E_{\max}\cdot C_{\mathrm{INH}}}{{\mathrm{EC}}_{50}+C_{\mathrm{INH}}},$$

(7) $$\mathrm{EFF}=\frac{E_{\max}\cdot {C_{\mathrm{INH}}}^{\gamma }}{{{\mathrm{EC}}_{50}}^{\gamma }+{C_{\mathrm{INH}}}^{\gamma }},$$

in which SLP represents the slope [(mg·L^−1^)^−1^], *E*_max_ the maximal response (−), EC_50_ the concentration of isoniazid responsible for 50% of the maximal response (mg·L^−1^), and *γ* the Hill exponent (−).

The exposure–response relationship was linked to the bacterial burden as either inhibition of growth or as kill term as shown for the exponential growth function as example in Equations 8 and 9, respectively.

(8) $$\frac{\text{dBac}}{\mathrm{dt}}=k_{g}\cdot \mathrm{Bac}\cdot \left(1-\mathrm{EFF}\right)$$

(9) $$\frac{\text{dBac}}{\mathrm{dt}}=k_{g}\cdot \mathrm{Bac}-\mathrm{EFF}\cdot \mathrm{Bac}$$

Biological (inter‐individual) variability was tested on the estimate of the inoculum, as well as on the parameters of the exposure–response relationship and reported as coefficient of variation. A proportional error model, parameterized as an additive error on the log_10_ transformed data, was used to describe the experimental (residual) variability in the bacterial burden.

Non‐linear mixed effects modelling does not assess statistical differences in outcome measures between treatment groups, rather it assesses whether inclusion of model features is statistically supported. In this framework, model selection was performed based on the likelihood ratio test between nested models, in which a drop in objective function value of 3.84 corresponds to *P* < 0.05 between models with a single degree of freedom difference, assuming a *χ* ^2^ distribution. Precision of the estimates of structural parameters was considered to be acceptable when relative standard errors of these estimates remained below 50%, which is a second means of assessing that the model is supported by the data. The physiological plausibility of parameter estimates and visual assessment of goodness‐of‐fit plots (Nguyen et al., 2017) were also used for model selection.

### 2.8 Translation of isoniazid response to humans

To quantitatively compare the findings for isoniazid response in zebrafish infected with *M. marinum* to findings in humans infected with *M. tuberculosis*, the exposure–response relationship obtained in the zebrafish larvae was translated to humans as a proof of concept.

For this, first, the isoniazid concentration–time profile in humans was simulated using a previously published pharmacokinetic model (Wilkins et al., 2011). Simulations for a period of 7 days of daily isoniazid oral dosing of 150, 300, and 450 mg for 1,000 individuals per dose group were simulated with a ratio of fast and slow metabolizers of 50:50.

Second, the simulated concentrations for the 1,000 individuals per dose group were linked to the exposure–response relationship for *M. marinum* in zebrafish larvae quantified here, to predict the isoniazid response on the bacterial burden. Two translational factors were utilized (Wicha et al., 2018). The difference in sensitivity to isoniazid between *M. marinum* and *M. tuberculosis* was taken into account by using the ratio of the MIC for *M marinum* and *M. tuberculosis* as a scaling factor for the slope (Schön et al., 2009). The difference in infection stage between the zebrafish, which is in logarithmic phase, and the patients, which are assumed to be in stationary phase (150 days of infection) (Wicha et al., 2018), was taken into account by scaling the isoniazid drug response as quantified previously (Clewe, Wicha, de Vogel, de Steenwinkel, & Simonsson, 2018). The ratio of the maximum isoniazid‐induced kill rates for the logarithmic and the stationary phase was utilized as translational factor to account for the difference in infection stage (Clewe et al., 2018). Inoculum of the simulation was set at the mean of the reported inoculi in humans (Hafner et al., 1997; Johnson et al., 2006; Li et al., 2010). The conversion between CFU·ml^−1^ and fluorescence as measures of infection was based on the known pathogen quantity of 200 CFU at time of injection and the fluorescence at time of injection estimated by the model (Supplementary material: Model code: Translation to human isoniazid response). The exponential growth rate of the bacterial burden was assumed to be similar, irrespective of measurement of bacterial burden by fluorescence (zebrafish) or CFU·ml^−1^ (humans).

Third, the obtained isoniazid response was quantitatively compared to published bacterial burden data in humans (Hafner et al., 1997; Johnson et al., 2006; Li et al., 2010).

### 2.9 Materials

Isoniazid, cinnamaldehyde, =tricaine and PVP40 were acquired from Sigma‐Aldrich and isoniazid‐D4 internal standard from Santa Cruz Biotechnology (Santa Cruz, USA). Nanopure water was used from a PURELAB water purification system (Veolia Water Technologies B.V., Ede, The Netherlands) unless otherwise stated. ULC/MS‐grade methanol as well as ULC/MS‐grade acetonitrile, LC/MS‐grade water, and formic acid was acquired from Biosolve B.V. (Valkenswaard, The Netherlands). Difco Middlebrook 7H10 agar and 7H9 medium, OADC, and ADC were acquired from Becton Dickinson and Company (Sparks, USA).

## 3 RESULTS

### 3.1 Internal exposure of isoniazid in zebrafish larvae

**FIGURE 2:**
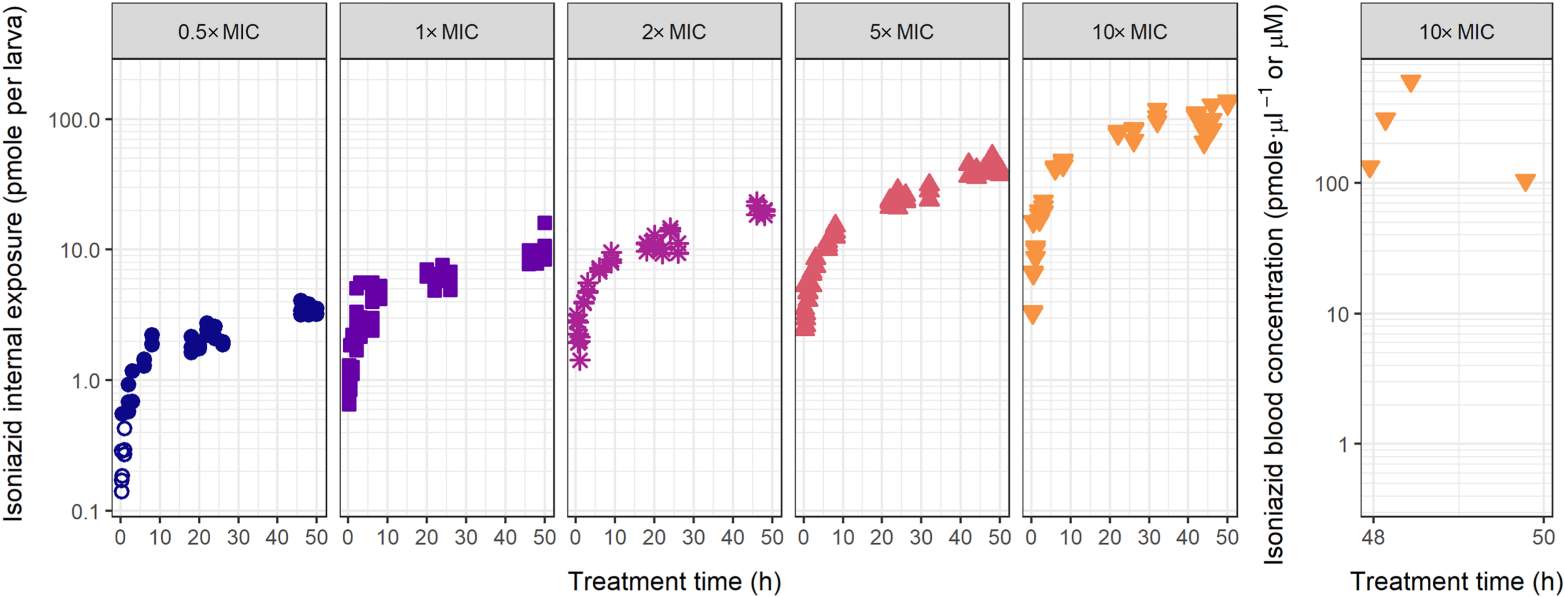
Internal isoniazid exposure over time in zebrafish larvae for increasing isoniazid doses. Internal exposure as pmol per larva in homogenate samples (left five panels) or as pmol·μl^−1^ in blood samples (right panel) is shown on a semi‐logarithmic scale for waterborne doses in the external treatment medium, from 0.5‐ to 10‐fold MIC, as indicated in each graph (MIC = 15 mg·L^−1^), for a constant treatment period of 50 h. Internal exposure linearly increases with dose, and steady state amounts increase with age, suggesting increased net absorption. Open symbols show observations below LLOQ

Internal exposure of isoniazid in zebrafish larvae after constant treatment between 3–5 dpf with increasing doses—defined here as external isoniazid concentration in the treatment medium—of 0.5, 1, 2, 5, and 10× MIC (7.5, 15, 30, 75, and 150 mg·L^−1^) was quantified. Figure 2 shows a clear linearity in exposure, where a 10‐fold higher dose resulted in a 10‐fold higher internal exposure. Internal exposure measured in homogenates reached steady state values within 12 h and after that increases with age.

To quantify the blood concentration in the zebrafish larvae of 5 dpf, an innovative novel blood sampling method was used. Median blood concentration of isoniazid at 48 h of treatment (5 dpf) with 150 mg·L^−1^ was 30.3 mg·L^−1^ (221 μM, range 14.4–82.6 mg·L^−1^, Figure 2), showing that the internal, blood concentration of isoniazid was only 20% of the external concentration.

### 3.2 *Mycobacterium marinum* bacterial burden in zebrafish larvae upon isoniazid treatment

**FIGURE 3:**
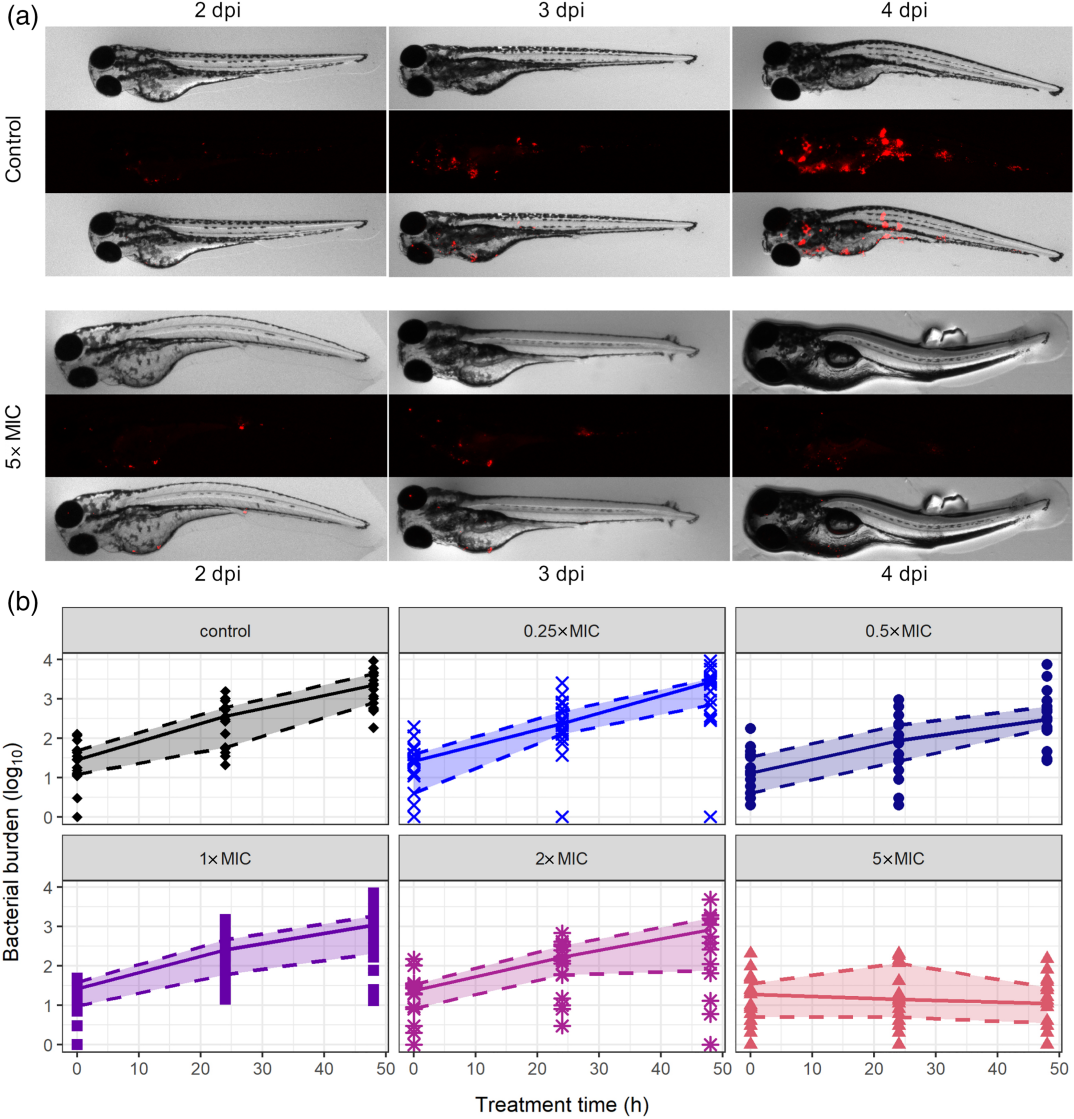
Bacterial burden in individual zebrafish larvae quantified by fluorescence imaging. (a) Representative images (brightfield [top], red fluorescence channel [middle], and overlay [bottom]) for control and 5‐fold MIC treatment groups at 2, 3, and 4 dpi (MIC = 15 mg·L^−1^). (b) The bacterial burden in fluorescent pixel count quantified by automated image analysis for control and treatment groups with doses 0.25–5× MIC (at least *n* = 17 individual larvae, measured daily). Symbols represent observations, while lines represent median and quantiles with the inter‐quantile range as shaded area

The bacterial burden of *M. marinum* was quantified through fluorescence imaging with repeated measurements of each individual larva. Doses ranging from 0.25 to 5× MIC were chosen based on a feasibility study and on the fluorescence detection limit. Figure 3 shows representative images of which the fluorescent pixels are quantified by automated image analysis software (Nezhinsky & Verbeek, 2010; Stoop et al., 2011) based on pixel counts. The median and interquartile range of the bacterial burden clearly show that bacterial growth is decreasing with increasing doses in comparison with the control. The highest dose of 5× MIC, which corresponded to 1× MIC blood concentration, showed a decline in the bacterial burden over time.

### 3.3 Quantification of the exposure–response relationship for isoniazid in zebrafish larvae

**FIGURE 4:**
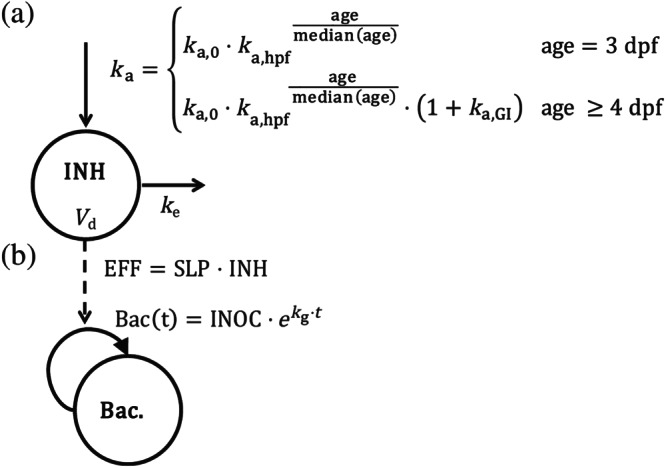
Schematic representation of the pharmacokinetic–pharmacodynamic model quantifying the internal exposure of isoniazid and its response on the bacterial burden in zebrafish larvae. Compartments represent (a) drug concentration or (b) bacterial count inside the larva, solid straight arrows represent pharmacokinetic mass transfer, the curved arrow represents bacterial growth, and the dashed arrow represents drug response. The top compartment shows the pharmacokinetic component of the model for isoniazid (INH) with a first‐order absorption rate constant (*k*_a_) from the external treatment medium, on which larval age is included as a covariate (Equation 10), distribution volume (*V*_d_), and first‐order elimination rate constant (*k*_e_). The bottom compartment shows the bacterial burden (Bac.) with exponential growth rate (*k*_g_) as growth function and inoculum at treatment time point zero (INOC). The exposure–response relationship (EFF) is quantified with a linear model with a slope (SLP). dpf, days post fertilization; GI, gastrointestinal; hpf, hours post fertilization; *k*_a,0_, absorption rate constant at the median age of 101 hours post fertilization (hpf); *k*_a,GI_, discrete factor with which the absorption rate constant increased at 4 days post fertilization (dpf) due to opening of the gastrointestinal tract; *k*_a,hpf_, constant in the exponential covariate relationship of age on absorption

To quantify the exposure–response relationship, a sequential modelling approach was performed. First, the internal exposure over time was quantified in the pharmacokinetic component of the model, after which this was linked to the isoniazid response in the final pharmacokinetic–pharmacodynamic model (Figure 4).

A one‐compartment model with first‐order absorption and first‐order elimination best described the data on internal exposure. Because the larvae are still developing, both absorption and elimination are expected to increase with age (Van Wijk, Krekels, Kantae, Harms, et al., 2019). The data show an increase in the steady state amounts with increasing age, suggesting that absorption rates increase faster than elimination rates. A net increase of absorption with age was found, which reflects a faster increase in absorption compared to the increase in elimination. Age was included as predictor (covariate) on the absorption rate constant (*k*_a_) in two ways: first, in an exponential relationship per hpf, and second, based on knowledge on the physiology of the GI tract, which opens between 3 and 4 dpf (Van Wijk, Krekels, Kantae, Harms, et al., 2019), as discrete increase at 4 dpf (Equation 10).

(10) $$k_{a}=\left\{\begin{array}{c} k_{a,0}\cdot k_{a,\mathrm{hpf}}^{\;\frac{\mathrm{age}}{\text{median}\left(\mathrm{age}\right)}}\;\;\mathrm{age}=3\;\mathrm{dpf}\\ k_{a,0}\cdot k_{a,\mathrm{hpf}}^{\;\frac{\mathrm{age}}{\text{median}\left(\mathrm{age}\right)}}\cdot \left(1+k_{a,\mathrm{GI}}\right)\;\;\mathrm{age}\geq 4\;\mathrm{dpf}\; \end{array}\right),$$

in which *k*_a,0_ is the absorption rate constant at the median age of 101 hpf, *k*_a,hpf_ is the constant in the exponential covariate relationship, and *k*_a,GI_ is the discrete factor with which the absorption rate constant increased at 4 dpf. A linear covariate relationship was statistically significantly worse (*P* < 0.001) compared to an exponential relationship, and a power relationship was statistically similar (*P* > 0.1) but resulted in worse precision of the parameter estimates.

**FIGURE 5:**
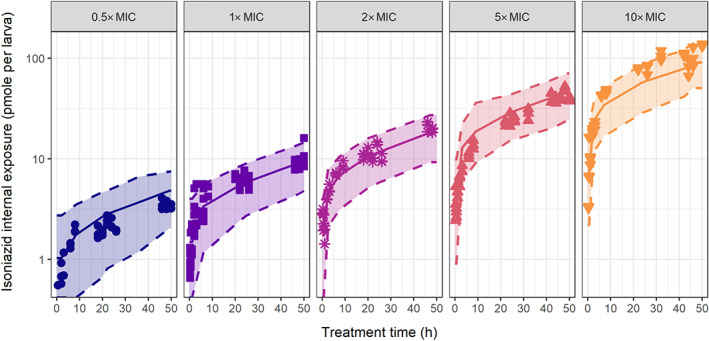
Model‐based prediction of the internal isoniazid exposure in zebrafish larvae of the final pharmacokinetic–pharmacodynamic model. Visual predictive check with median (solid line) and 95% prediction interval (dashed lines, shaded area) from 500 simulations based on the pharmacokinetic component of the final model shows good prediction of the observed data (symbols, at least *n* = 3 with five larvae per sample) of internal exposure obtained after constant waterborne isoniazid treatment of 0.5‐ to 10‐fold MIC as indicated in each graph (MIC = 15 mg·L^−1^)

Precision of all pharmacokinetic parameters in the final model was acceptable (relative standard errors <36%), with only the relative standard error of *k*_a,GI_ being slightly higher than the cut‐off of 50%. Removing the effect of the opening of the GI tract worsened the fit significantly (*P* < 0.05), and as the opening of the GI tract was physiologically expected to affect absorption, the relationship was retained despite the slight imprecision of the obtained estimate. The precision of the remaining model parameters confirmed that the obtained model and parameter values are supported by the data. Goodness‐of‐fit plots further confirm an unbiased fit of the data by the model (Figure S2). A visual predictive check is provided in Figure 5, which showed good prediction of the typical trends and a slight over‐prediction of the variability of the observed data by the model.

**FIGURE 6:**
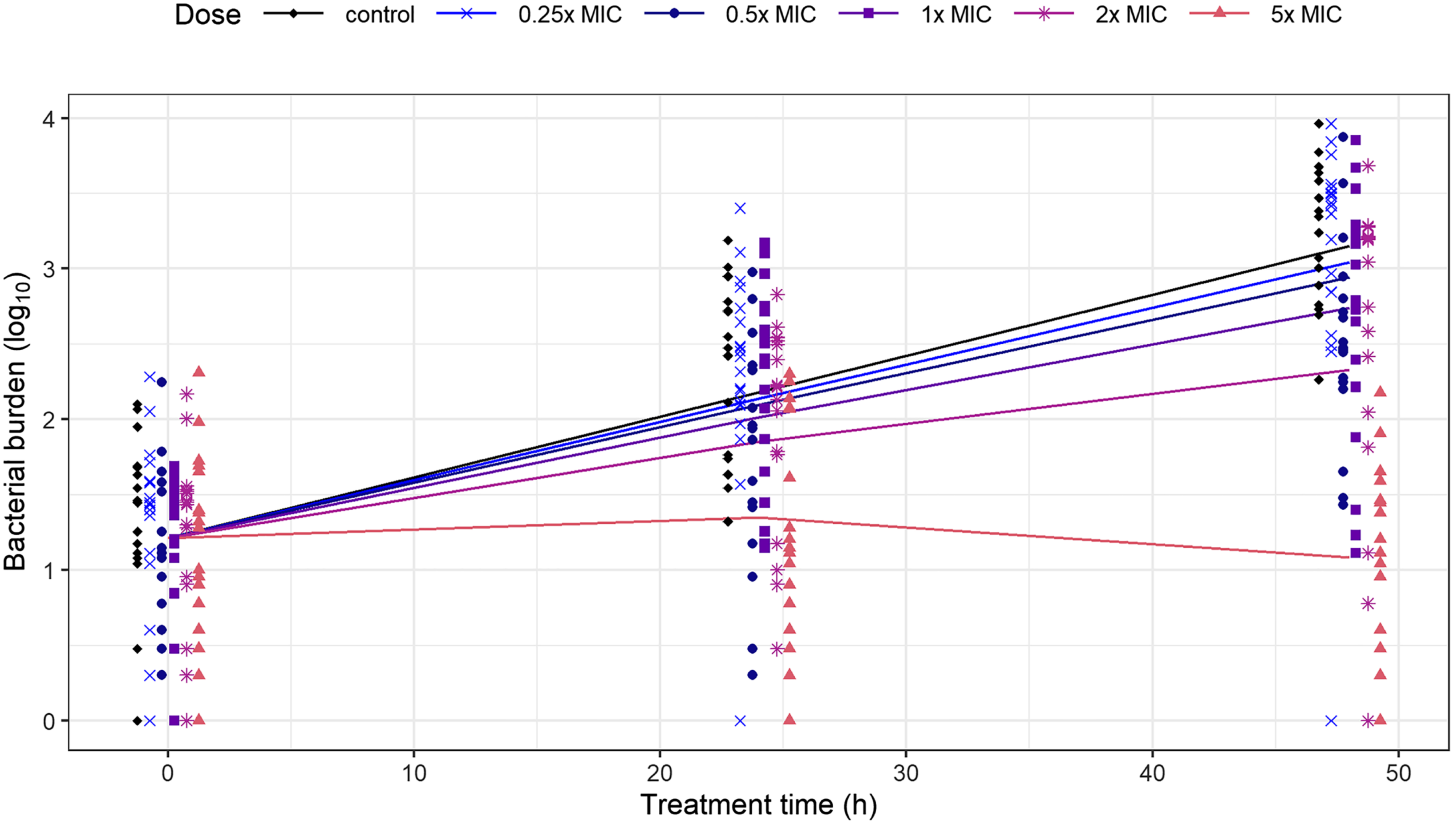
Observed and model‐based prediction of the bacterial burden after isoniazid treatment in individual zebrafish larvae infected with *Mycobacterium marinum*. The bacterial burden as log_10_‐transformed fluorescent pixel count is shown over treatment time of 50 h at isoniazid doses in the external treatment medium of 0.25×, 0.5×, 1×, 2×, and 5‐fold MIC, in addition to control. Symbols represent observed data (at least *n* = 17 individual larvae, measured daily), and lines connect model predictions. Biological variability as quantified by the model is relatively large in contrast to experimental variability (see Figure S3 for individual predictions)

The data of bacterial burden did not support separate estimation of growth and decay; therefore, only the net effect of these two processes was estimated in the model, which was found to be best described with an exponential growth model. In case of bacterial kill exceeding bacterial growth with sufficient isoniazid response, this net growth rate would become negative. A linear exposure–response relationship for inhibition of bacterial growth fitted the data best. The biological variability between larvae was quantified by inclusion of inter‐individual variability on the inoculum (coefficient of variation 204%) and slope of the drug response (coefficient of variation 50.5%). The experimental variability was very reasonable with a coefficient of variation of 36.3%. The predicted isoniazid response in zebrafish larvae is shown in Figure 6 (individual predictions: Figure S3), with a clear increase in antibacterial response with increasing external concentrations, which is in line with the observations. Goodness‐of‐fit plots show unbiased model fit (Figure S4). Parameter estimates of the final pharmacokinetic–pharmacodynamic model are given in Table 1.

**TABLE 1:** Parameter estimates of the final pharmacokinetic–pharmacodynamic model as shown in Figure 4

Parameter	Estimate	Relative standard error of estimate (%)
Structural parameters pharmacokinetic component		
*k*_a,0_ (μl·h^−1^)	0.00349	25
*k*_a,hpf_ (−)	7.61	17
*k*_a,GI_ (−)	0.171	51
*k*_e_ (h^−1^)	0.580	32
*V*_d_ (μl)	0.325	36
Structural parameters pharmacodynamic component		
*k*_g_ (h^−1^)	0.0930	4
INOC (Fluorescence)	16.3	13
SLP (μM^−1^)	0.00991	37
Stochastic parameters pharmacodynamic component		
Variance (*ω* ^2^) of biological variability INOC (−)	1.64	18
Variance (*ω* ^2^) of biological variability SLP (−)	0.227	93
Residual experimental error pharmacokinetic component		
Variance of proportional experimental error homogenate (−)	0.0609	24
Variance of additive experimental error homogenate (pmol·larva^−1^)	0.591	45
Variance of proportional experimental error blood (−)	0.482	49
Residual experimental error pharmacodynamic component		
Variance of proportional experimental error (−)	0.124	17

Abbreviations: INOC, inoculum at treatment time point zero; *k*_a,0_, absorption rate constant at the median age of 101 hours post fertilization (hpf); *k*_a,GI_, discrete factor with which the absorption rate constant increased at 4 days post fertilization (dpf) due to opening of the gastrointestinal tract; *k*_a,hpf_, constant in the exponential covariate relationship of age on absorption; *k*_e_, first‐order elimination rate constant; *k*_g_, mycobacterial growth rate constant; SLP, slope of drug effect; *V*_d_, volume of distribution.

### 3.4 Translation of isoniazid response to humans

An exposure–response relationship of a drug is often assumed to be conserved between vertebrates (Bartelink et al., 2017; De Groote et al., 2011; Van Wijk, Ayoun Alsoud, et al., 2020; Wicha et al., 2018). As a proof of concept for the translation of the quantified exposure–response relationship in zebrafish larvae to humans, the relationship for isoniazid in zebrafish larvae was translated to humans, assuming the isoniazid response of *M. tuberculosis* in humans to be similar to the isoniazid response of its close relative *M. marinum* in the larvae. Two translational factors were taken into account (Wicha et al., 2018). The first corrected for the difference in sensitivity to isoniazid between *M. marinum* and *M. tuberculosis* as reported by a difference in MIC. The second corrected for the difference in stage of infection between the fresh experimental infection and more chronic clinical infection, where it was assumed that patients start treatment 150 days after initial infection (Wicha et al., 2018).

**FIGURE 7:**
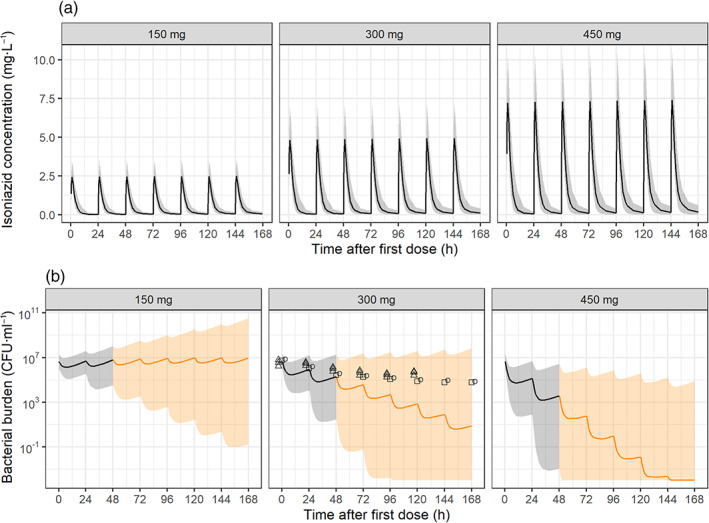
Translation of isoniazid response in zebrafish larvae to humans, using a model‐based pharmacokinetic–pharmacodynamic approach. (a) isoniazid concentration–time profile (median: solid line, 80% prediction interval: shaded area) after 7 days of daily isoniazid doses of 150, 300, and 450 mg as simulated from a previously published pharmacokinetic model (Wilkins et al., 2011). (b) Simulated median (solid line) and 80% prediction interval (shaded area, including biological and experimental variability) bacterial burden in CFU·ml^−1^ sputum based on the human isoniazid concentration–time profile for 1,000 individuals per dose group. Concentration–time profiles were linked to the exposure–response relationship quantified in zebrafish larvae together with the translational factors on isoniazid sensitivity (MIC) and stage of infection (logarithmic vs. stationary). Translated response corresponds well to the observed bacterial burden in sputum, from Hafner et al., (1997; triangles), Johnson et al., (2006; squares) and Li et al., (2010; circles). Orange part of the prediction is extrapolated in time from the 48 h of treatment studied in the zebrafish, shown in black

The exposure–response relationship quantified in the current study was linked to simulated isoniazid concentration–time profiles using a previously published pharmacokinetic model for isoniazid in humans (Wilkins et al., 2011). Simulations with three human doses were performed: a subtherapeutic and a super therapeutic dose of 150 and 450 mg, in addition to the recommended dose of 300 mg. Reported observations in patients of *M. tuberculosis* bacterial burden quantified in sputum after isoniazid monotherapy of daily doses of 300 mg (Hafner et al., 1997; Johnson et al., 2006; Li et al., 2010) served as quantitative comparison for our simulated isoniazid effect. Figure 7 shows the simulated concentration–time profile for virtual patients (median and 80% prediction interval, which includes biological and experimental variability) and the bacterial burden–time profiles (median and 80% prediction interval, which includes biological and experimental variability) per dose group, the latter of which for a dose of 300 mg was in good agreement with the observed data. The simulations after subtherapeutic dose of 150 mg showed limited isoniazid response and hence bacterial growth in the majority of the population. The therapeutic dose of 300 mg showed a decline in the median bacterial burden. The median translated bacterial burden declined 5–6 log_10_ CFU·ml^−1^ during the 7 days of treatment period or 0.7–0.9 log_10_ CFU·ml^−1^·day^−1^. The super therapeutic dose of 450 mg showed a decline in bacterial burden in more than 80% of the patients and on average a steeper decline of the bacterial burden than the therapeutic dose.

The translated isoniazid response at the therapeutic dose correlated well with the observed data for the first 48 h, which was also the duration of treatment studied in the zebrafish larvae in this work. Extrapolating to later time points showed a slight over‐prediction of isoniazid response as evident by the steeper decline in bacterial burden than the observed data, while the observations were still within the prediction interval. This proof of concept suggests that the zebrafish larva is a promising addition to the quantitative model‐based translational pipeline in anti‐TB drug development.

## 4 DISCUSSION

There is a clear need for innovation in anti‐TB drug development. With its high‐throughput potential, its possibility for repeated fluorescence imaging to quantify infection, and its fast, cheap, and relatively safe experimentation, the zebrafish larva tuberculosis disease model combines the advantages of *in vitro* experiments within a whole‐organism vertebrate with its translational potential. Additionally, zebrafish embryos and larvae prior to independent feeding are not considered experimental animals, and no approval of an ethical committee is required for these experiments (EU, 2010; Strähle et al., 2012). In this work, we developed an experimental approach to acquire data on the internal drug exposure and on the bacterial burden over time. We utilized computational pharmacokinetic–pharmacodynamic modelling to quantify the exposure–response relationship of isoniazid in zebrafish larvae. Based on that quantitative pharmacological relationship and two translational factors, early bactericidal isoniazid response in TB treatment could successfully be translated to humans. This proof‐of‐concept translation to human response shows the strengths of quantifying internal exposure–response relationships in zebrafish larvae and its application to translate pharmacological findings to humans, a seemingly different organism. Using our innovative workflow, zebrafish studies can improve quantitative understanding of *in vivo* drug response early in the drug development process. Through this quantitative model‐based approach, the zebrafish larva becomes a full member of the translational pipeline in drug development.

Quantification of internal drug exposure in zebrafish larvae after external, waterborne drug treatment is essential for reliable interpretation of drug response, but because of small sample volumes and low concentrations, this is not straightforward. We have built upon recent work to develop an ultra‐sensitive LC–MS based quantification method (Kantae et al., 2016; Van Wijk, Krekels, Kantae, Harms, et al., 2019; Van Wijk, Krekels, Kantae, Ordas, et al., 2019). This methodology proved sensitive enough to quantify isoniazid in larval homogenates and blood samples of only nanolitres in volume. The latter is especially of importance because blood concentrations are required to scale total amounts in homogenates to blood concentrations, which is essential for establishing a drug concentration–effect relationship, which is the basis for inter‐species translation. The state‐of‐the‐art blood sampling method cannot yet be performed in high throughput mode, but automation based on previously developed automated injection systems is being developed (Spaink et al., 2013).

Utilizing fluorescence imaging in transparent larvae to establish the response of mycobacterial burden to isoniazid has clear advantages over CFU plating, as the latter has been reported to show large sample‐to‐sample variability (Gillespie, Gosling, & Charalambous, 2002). An important advantage of fluorescence imaging in zebrafish larvae is the possibility of repeated longitudinal measurements of the bacterial burden within a single individual, which is uncommon in preclinical TB research. These repeated measurements not only suppress noise by distinguishing biological from experimental variability but also reduce the number of subjects needed in an experiment, which is ethically preferable. Additionally, fluorescence imaging of bacteria does not require these bacteria to grow on solid or in liquid media and will include both multiplying and non‐multiplying (i.e., dormant) bacteria. Currently, our imaging set‐up is restricted by its fluorescence detection limit to clearly quantify mycobacterial kill, which is why the highest dose of 10× MIC was not tested in the bacterial burden study. Imaging systems are continuously being improved, pushing the detection limit to the individual mycobacterium (Greenwood et al., 2019).

\*Mycobacterium marinum* and *M. tuberculosis* show different sensitivities to isoniazid. Isoniazid MIC against *M. marinum* ranges between 1.6 and 32 mg·L^−1^ (Aubry et al., 2000; Boot et al., 2016; Boot et al., 2017; Weerakhun, Hatai, Murase, & Hirae, 2008), in concordance with the value obtained in our analysis. The MIC against *M. tuberculosis* is lower and ranges from 0.016 to 0.2 mg·L^−1^ (Budha, Lee, Hurdle, Lee, & Meibohm, 2009; Gumbo et al., 2007; Hemanth Kumar et al., 2016; Jayaram et al., 2004; Schön et al., 2009). This difference in sensitivity was taken into account when predicting isoniazid response in humans by scaling the slope with the ratio of MICs as the scaling term, as shown earlier as a method for handling differences in sensitivities to drugs between strains (Wicha et al., 2018). The difference in stage of infection between the logarithmic growth upon a fresh experimental infection in the zebrafish here and the chronic, stationary infection of patients starting treatment was taken into account as well. This translational factor has been presented before and is important to consider when translating results from a well‐controlled experimental context to the real world (Wicha et al., 2018).

The pharmacokinetic–pharmacodynamic model developed here was data driven. The internal exposure over time could be described well by the pharmacokinetic component of the model, although the effects of age on absorption and elimination could not be quantified separately. Translation from zebrafish larvae to humans based on this empirical model was good and shows the translational potential of the zebrafish larva as a new disease model in tuberculosis drug development. The experimental and modelling approach presented here in the zebrafish larva would yield insight into the exposure–response of the drug under development, which will improve informed decision making. The translation from zebrafish larvae to higher vertebrates will further improve with the addition of more physiological or mechanistic details. A multistate tuberculosis pharmacometric (MTP) model has been developed previously for *M. tuberculosis*, quantifying *in vitro* natural growth over 200 days using a fast‐, slow‐, and non‐multiplying subpopulation. The drug responses of isoniazid, rifampicin and ethambutol have been quantified on these states (Clewe, Aulin, Hu, Coates, & Simonsson, 2016) and successfully translated to mice (Chen et al., 2017) and patients (Clewe et al., 2018; Svensson & Simonsson, 2016). This multistate approach, especially when the *in vitro* natural growth and time‐kill is repeated for *M. marinum* as well, can be integrated with our analysis of *M. marinum* to strengthen the translational value of findings in zebrafish to higher vertebrates (Van Wijk, Van der Sar, et al., 2020).

The reported bacterial burden in humans after daily 300mg isoniazid monotherapy fell within the 80% prediction interval (including biological and experimental variability) obtained from simulated concentration–time profiles in humans and the exposure–response relationship quantified in zebrafish larvae here. The median simulated decline in the bacterial burden of 0.7–0.9 log_10_ CFU·ml^−1^·day^−1^ after 2 days of treatment, has been reported in humans before (Jindani, Doré, & Mitchison, 2003). The large variability in the prediction interval from this work was largely due to the high biological variability in the inoculum, resulting from the establishment of infection during the first 2 days and the slope of the linear drug response quantified in zebrafish larva. Care must also be taken when extrapolating a linear exposure–response relationship to exposures outside the studied range, as well as extrapolating the response of treatment after the 48 h of treatment in zebrafish larvae. The treatment duration in the zebrafish larvae was not extended beyond 48 h, to remain within the ethical larval age limit (Figure 1) (Strähle et al., 2012).

In conclusion, we have developed a new experimental and computational approach to translate the pharmacokinetic–pharmacodynamic relationship of the early bactericidal response to an antibiotic in a zebrafish model of TB to humans. We propose that this approach is used in the search for novel TB treatments.

## AUTHOR CONTRIBUTIONS

R.C.v.W., U.S.H.S., H.P.S., P.H.v.d.G., and E.H.J.K. contributed to the conceptualization of this study; R.C.v.W., W.H., and S.M.D. contributed to the investigation; J.L., R.B., D.J.v.d.B., and F.J.V. contributed to the methodology; R.C.v.W. and E.H.J.K. contributed to the formal analysis and writing of the original draft; and W.H., S.M.D., J.L.,. R.B., D.J.v.d.B., F.J.V., U.S.H.S., H.P.S., and P.H.v.d.G. contributed to the writing (review and editing) of the paper.

## CONFLICT OF INTEREST

The authors declare no conflicts of interest.

## DECLARATION OF TRANSPARENCY AND SCIENTIFIC RIGOUR

This Declaration acknowledges that this paper adheres to the principles for transparent reporting and scientific rigour of preclinical research as stated in the *BJP* guidelines for Design and Analysis, and as recommended by funding agencies, publishers and other organisations engaged with supporting research.

## Supporting information


**Figure S1.** Goodness‐of‐fit plots for the pharmacokinetic component of the final pharmacokinetic‐pharmacodynamic model in zebrafish larvae.
Figure S2. Model‐based individual prediction of the bacterial burden after isoniazid treatment in zebrafish larvae infected with *M. marinum*
Figure S3. Goodness‐of‐fit plots for the pharmacodynamic component of the final pharmacokinetic‐pharmacodynamic model in zebrafish larvae.
Figure S4. Stability of waterborne isoniazid over treatment period.
Table S1. Overview of the blood sampling experiment, reporting the number of larvae sampled per timepoint, the total blood volume, and the isoniazid concentration.
Model code: Final pharmacokinetic‐pharmacodynamic model in zebrafish infected with *M. marinum*
Model code: Translation to human isoniazid response

## Data Availability

Final model codes and data sets are publicly available through the DDMoRe Repository, Model ID DDMODEL00000311 (http://repository.ddmore.foundation/model/DDMODEL00000311).

## References

[bph15247-bib-0001] Aubry, A. , Jarlier, V. , Escolano, S. , Truffot‐Pernot, C. , & Cambau, E. (2000). Antibiotic susceptibility pattern of *Mycobacterium marinum* . Antimicrobial Agents and Chemotherapy, 44, 3133–3136. 10.1128/AAC.44.11.3133-3136.2000 11036036 PMC101616

[bph15247-bib-0002] Bartelink, I. H. , Zhang, N. , Keizer, R. J. , Strydom, N. , Converse, P. J. P. , Dooley, K. E. K. , et al. (2017). New paradigm for translational modeling to predict long‐term tuberculosis treatment response. Clinical and Translational Science, 10, 366–379. 10.1111/cts.12472 28561946 PMC5593171

[bph15247-bib-0003] Beal, S. , Sheiner, L. , Boeckmann, A. , & Bauer, R. J. (2013). NONMEM 7.3.0 users guides. (1989–2013) In ICON development solutions. MD, USA: Hanover.

[bph15247-bib-0004] Beal, S. L. (2001). Ways to fit a PK model with some data below the quantification limit. Journal of Pharmacokinetics and Pharmacodynamics, 28, 481–504. 10.1023/A:1012299115260 11768292

[bph15247-bib-0005] Benard, E. L. , Van Der Sar, A. M. , Ellett, F. , Lieschke, G. J. , Spaink, H. P. , & Meijer, A. H. (2012). Infection of zebrafish embryos with intracellular bacterial pathogens. Journal of Visualized Experiments, 61, e3781 10.3791/3781 PMC3415172 22453760

[bph15247-bib-0006] Boot, M. , Jim, K. K. , Liu, T. , Commandeur, S. , Lu, P. , Verboom, T. , et al. (2017). A fluorescence‐based reporter for monitoring expression of mycobacterial cytochrome bd in response to antibacterials and during infection. Scientific Reports, 7, 1–10. 10.1038/s41598-017-10944-4 28878275 PMC5587683

[bph15247-bib-0007] Boot, M. , Sparrius, M. , Jim, K. K. , Commandeur, S. , Speer, A. , Van De Weerd, R. , et al. (2016). iniBAC induction is vitamin B12‐ and MutAB‐dependent in *Mycobacterium marinum* . The Journal of Biological Chemistry, 291, 19800–19812. 10.1074/jbc.M116.724088 27474746 PMC5025670

[bph15247-bib-0008] Budha, N. R. , Lee, R. B. , Hurdle, J. G. , Lee, R. E. , & Meibohm, B. (2009). A simple in vitro PK/PD model system to determine time‐kill curves of drugs against Mycobacteria. Tuberculosis, 89, 378–385. 10.1016/j.tube.2009.08.002 19748318 PMC2783979

[bph15247-bib-0009] Carvalho, R. , de Sonneville, J. , Stockhammer, O. W. , Savage, N. D. L. , Veneman, W. J. , Ottenhoff, T. H. M. , et al. (2011). A high‐throughput screen for tuberculosis progression. PLoS ONE, 6, 1–8. 10.1371/journal.pone.0016779 PMC3040195 21390204

[bph15247-bib-0010] Chen, C. , Ortega, F. , Rullas, J. , Alameda, L. , Angulo‐Barturen, I. , Ferrer, S. , & Simonsson, U. S. H. (2017). The multistate tuberculosis pharmacometric model: A semi‐mechanistic pharmacokinetic‐pharmacodynamic model for studying drug effects in an acute tuberculosis mouse model. Journal of Pharmacokinetics and Pharmacodynamics, 44, 133–141. 10.1007/s10928-017-9508-2 28205025 PMC5376397

[bph15247-bib-0011] Clewe, O. , Aulin, L. , Hu, Y. , Coates, A. R. M. , & Simonsson, U. S. H. (2016). A multistate tuberculosis pharmacometric model: A framework for studying anti‐tubercular drug effects in vitro. The Journal of Antimicrobial Chemotherapy, 71, 964–974. 10.1093/jac/dkv416 26702921 PMC4790616

[bph15247-bib-0012] Clewe, O. , Wicha, S. G. , de Vogel, C. P. , de Steenwinkel, J. E. M. , & Simonsson, U. S. H. (2018). A model‐informed preclinical approach for prediction of clinical pharmacodynamic interactions of anti‐TB drug combinations. The Journal of Antimicrobial Chemotherapy, 73, 437–447. 10.1093/jac/dkx380 29136155 PMC5890720

[bph15247-bib-0013] De Groote, M. A. , Gilliland, J. C. , Wells, C. L. , Brooks, E. J. , Woolhiser, L. K. , Gruppo, V. , et al. (2011). Comparative studies evaluating mouse models used for efficacy testing of experimental drugs against *Mycobacterium tuberculosis* . Antimicrobial Agents and Chemotherapy, 55, 1237–1247. 10.1128/AAC.00595-10 21135176 PMC3067068

[bph15247-bib-0014] DiMasi, J. A. , Grabowski, H. G. , & Hansen, R. W. (2016). Innovation in the pharmaceutical industry: New estimates of R&D costs. Journal of Health Economics, 47, 20–33. 10.1016/j.jhealeco.2016.01.012 26928437

[bph15247-bib-0015] European Union . (2010). Council directive 2010/63/EU on the protection of animals used for scientific purposes. Official Journal of the European Union, L276(33).

[bph15247-bib-0016] Furin, J. , Cox, H. , & Pai, M. (2019). Tuberculosis. Lancet, 393, 1642–1656. 10.1016/S0140-6736(19)30308-3 30904262

[bph15247-bib-0017] Gillespie, S. H. , Gosling, R. D. , & Charalambous, B. M. (2002). A reitrerative method for calculating the early bactericidal activity of antituberculosis drugs. American Journal of Respiratory and Critical Care Medicine, 166, 31–35. 10.1164/rccm.2112077 12091167

[bph15247-bib-0018] Ginsberg, A. M. , & Spigelman, M. (2007). Challenges in tuberculosis drug research and development. Nature Medicine, 13, 290–294. 10.1038/nm0307-290 17342142

[bph15247-bib-0019] Greenwood, D. J. , Santos, M. S. D. , Huang, S. , Russell, M. R. G. , Collinson, L. M. , MacRae, J. I. , et al. (2019). Subcellular antibiotic visualization reveals a dynamic drug reservoir in infected macrophages. Science, 364(80), 1279–1282. 10.1126/science.aat9689 31249058 PMC7012645

[bph15247-bib-0020] Gumbo, T. , Louie, A. , Liu, W. , Brown, D. , Ambrose, P. G. , Bhavnani, S. M. , & Drusano, G. L. (2007). Isoniazid bactericidal activity and resistance emergence: Integrating pharmacodynamics and pharmacogenomics to predict efficacy in different ethnic populations. Antimicrobial Agents and Chemotherapy, 51, 2329–2336. 10.1128/AAC.00185-07 17438043 PMC1913269

[bph15247-bib-0021] Guo, Y. , Veneman, W. J. , Spaink, H. P. , & Verbeek, F. J. (2017). Three‐dimensional reconstruction and measurements of zebrafish larvae from high‐throughput axial‐view in vivo imaging. Biomedical Optics Express, 8, 2611–2634. 10.1364/BOE.8.002611 28663894 PMC5480501

[bph15247-bib-0022] Hafner, R. , Cohn, J. A. , Wright, D. J. , Dunlap, N. E. , Egorin, M. J. , Enama, M. E. , et al. (1997). Early bactericidal activity of isoniazid in pulmonary tuberculosis: Optimization of methodology. American Journal of Respiratory and Critical Care Medicine, 156, 918–923. 10.1164/ajrccm.156.3.9612016 9310014

[bph15247-bib-0023] Hemanth Kumar, A. K. , Kannan, T. , Chandrasekaran, V. , Sudha, V. , Vijayakumar, A. , Ramesh, K. , … Ramachandran, G. (2016). Pharmacokinetics of thrice‐weekly rifampicin, isoniazid and pyrazinamide in adult tuberculosis patients in India. The International Journal of Tuberculosis and Lung Disease, 20, 1236–1241. 10.5588/ijtld.16.0048 27510252

[bph15247-bib-0024] Jayaram, R. , Shandil, R. K. , Gaonkar, S. , Kaur, P. , Suresh, B. L. , Mahesh, B. N. , … Balasubramanian, V. (2004). Isoniazid pharmacokinetics–pharmacodynamics in an aerosol infection model of tuberculosis. Antimicrobial Agents and Chemotherapy, 48, 2951–2957. 10.1128/AAC.48.8.2951-2957.2004 15273105 PMC478500

[bph15247-bib-0025] Jindani, A. , Aber, V. R. , Edwards, E. A. , & Mitchison, D. A. (1980). The early bactericidal activity of drugs in patients with pulmonary tuberculosis. The American Review of Respiratory Disease, 121, 939–949. 10.1164/arrd.1980.121.6.939 6774638

[bph15247-bib-0026] Jindani, A. , Doré, C. J. , & Mitchison, D. A. (2003). Bactericidal and sterilizing activities of antituberculosis drugs during the first 14 days. American Journal of Respiratory and Critical Care Medicine, 167, 1348–1354. 10.1164/rccm.200210-1125OC 12519740

[bph15247-bib-0027] Johnson, J. L. , Hadad, D. J. , Boom, W. H. , Daley, C. L. , Peloquin, C. A. , Eisenach, K. D. , … Dietze, R. (2006). Early and extended early bactericidal activity of levofloxacin, gatifloxacin and moxifloxacin in pulmonary tuberculosis. The International Journal of Tuberculosis and Lung Disease, 10, 605–612. 16776446

[bph15247-bib-0028] Kantae, V. , Krekels, E. H. J. , Ordas, A. , González, O. , Van Wijk, R. C. , Harms, A. C. , et al. (2016). Pharmacokinetic modeling of paracetamol uptake and clearance in zebrafish larvae: Expanding the allometric scale in vertebrates with five orders of magnitude. Zebrafish, 13, 504–510. 10.1089/zeb.2016.1313 27632065 PMC5124745

[bph15247-bib-0029] Keizer, R. , Van Benten, M. , Beijnen, J. , Schellens, J. , & Huitema, A. (2011). Pirana and PCluster: A modeling environment and cluster infrastructure for NONMEM. Computer Methods and Programs in Biomedicine, 101, 72–79. 10.1016/j.cmpb.2010.04.018 20627442

[bph15247-bib-0030] Kolibab, K. , Yang, A. , Parra, M. , Derrick, S. C. , & Morris, S. L. (2014). Time to detection of *Mycobacterium tuberculosis* using the MGIT 320 system correlates with colony counting in preclinical testing of new vaccines. Clinical and Vaccine Immunology, 21, 453–455. 10.1128/CVI.00742-13 24371256 PMC3957671

[bph15247-bib-0031] Kumar, N. , Vishwas, K. G. , Kumar, M. , Reddy, J. , Parab, M. , Manikanth, C. L. , … Shandil, R. K. (2014). Pharmacokinetics and dose response of anti‐TB drugs in rat infection model of tuberculosis. Tuberculosis, 94, 282–286. 10.1016/j.tube.2014.02.004 24629633

[bph15247-bib-0032] Li, L. , Mahan, C. S. , Palaci, M. , Horter, L. , Loeffelholz, L. , Johnson, J. L. , … Eisenach, K. D. (2010). Sputum *Mycobacterium tuberculosis* mRNA as a marker of bacteriologic clearance in response to antituberculosis therapy. Journal of Clinical Microbiology, 48, 46–51. 10.1128/JCM.01526-09 19923475 PMC2812283

[bph15247-bib-0033] Lindbom, L. , Pihlgren, P. , & Jonsson, E. (2005). PsNtoolkit—A collection of computer intensive statistical methods for non‐linear mixed effect modeling using NONMEM. Computer Methods and Programs in Biomedicine, 79, 241–257. 10.1016/j.cmpb.2005.04.005 16023764

[bph15247-bib-0034] Meijer, A. H. (2016). Protection and pathology in TB: Learning from the zebrafish model. Seminars in Immunopathology, 38, 261–273. 10.1007/s00281-015-0522-4 26324465 PMC4779130

[bph15247-bib-0035] Meijer, A. H. , & Spaink, H. P. (2011). Host–pathogen interactions made transparent with the zebrafish model. Curr. Drug Targets, 12, 1000–1017. 10.2174/138945011795677809 PMC3319919 21366518

[bph15247-bib-0036] Morgan, P. , van der Graaf, P. H. , Arrowsmith, J. , Feltner, D. E. , Drummond, K. S. , Wegner, C. D. , et al. (2012). Can the flow of medicines be improved? Fundamental pharmacokinetic and pharmacological principles toward improving phase II survival. Drug Discovery Today, 17, 419–424. 10.1016/j.drudis.2011.12.020 22227532

[bph15247-bib-0037] Nezhinsky, A. , & Verbeek, F. J. (2010). Pattern recognition for high throughput zebrafish imaging using genetic algorithm optimization In Dijkstra T., Tsivtsivadze E., Heskes T., & Marchiori E. (Eds.), Lecture notes in bioinformatics 6282 (pp. 301–312). Berlin ‐ Heidelberg: Springer‐Verlag 10.1007/978-3-642-16001-1_26

[bph15247-bib-0038] Nguyen, T. H. T. , Mouksassi, M.‐S. , Holford, N. , Al‐Huniti, N. , Freedman, I. , Hooker, A. , et al. (2017). Model evaluation of continuous data pharmacometric models: Metrics and graphics. CPT: Pharmacometrics & Systems Pharmacology, 6, 87–109. 10.1002/psp4.12161 27884052 PMC5321813

[bph15247-bib-0039] Ordas, A. , Raterink, R.‐J. , Cunningham, F. , Jansen, H. J. , Wiweger, M. I. , Jong‐Raadsen, S. , … Spaink, H. P. (2015). Testing tuberculosis drug efficacy in a zebrafish high‐throughput translational medicine screen. Antimicrobial Agents and Chemotherapy, 59, 753–762. 10.1128/AAC.03588-14 25385118 PMC4335901

[bph15247-bib-0040] Rennekamp, A. J. , & Peterson, R. T. (2015). 15 years of zebrafish chemical screening. Current Opinion in Chemical Biology, 24, 58–70. 10.1016/j.cbpa.2014.10.025 25461724 PMC4339096

[bph15247-bib-0041] Schön, T. , Juréen, P. , Giske, C. G. , Chryssanthou, E. , Sturegård, E. , Werngren, J. , et al. (2009). Evaluation of wild‐type MIC distributions as a tool for determination of clinical breakpoints for *Mycobacterium tuberculosis* . The Journal of Antimicrobial Chemotherapy, 64, 786–793. 10.1093/jac/dkp262 19633001

[bph15247-bib-0042] Schulthess, P. , Van Wijk, R. C. , Krekels, E. H. J. , Yates, J. W. T. , Spaink, H. P. , & Van der Graaf, P. H. (2018). Outside‐in systems pharmacology combines innovative computational methods with high‐throughput whole vertebrate studies. CPT: Pharmacometrics & Systems Pharmacology, 7, 285–287. 10.1002/psp4.12297 29693322 PMC5980533

[bph15247-bib-0043] Spaink, H. P. , Cui, C. , Wiweger, M. I. , Jansen, H. J. , Veneman, W. J. , Marín‐Juez, R. , … Dirks, R. P. (2013). Robotic injection of zebrafish embryos for high‐throughput screening in disease models. Methods, 62, 246–254. 10.1016/j.ymeth.2013.06.002 23769806

[bph15247-bib-0044] Stoop, E. J. M. , Schipper, T. , Rosendahl Huber, S. K. , Nezhinsky, A. E. , Verbeek, F. J. , Gurcha, S. S. , … Van der Sar, A. M. (2011). Zebrafish embryo screen for mycobacterial genes involved in the initiation of granuloma formation reveals a newly identified ESX‐1 component. Disease Models & Mechanisms, 4, 526–536. 10.1242/dmm.006676 21372049 PMC3124061

[bph15247-bib-0045] Strähle, U. , Scholz, S. , Geisler, R. , Greiner, P. , Hollert, H. , Rastegar, S. , … Braunbeck, T. (2012). Zebrafish embryos as an alternative to animal experiments—A commentary on the definition of the onset of protected life stages in animal welfare regulations. Reproductive Toxicology, 33, 128–132. 10.1016/j.reprotox.2011.06.121 21726626

[bph15247-bib-0046] Svensson, R. J. , & Simonsson, U. S. H. (2016). Application of the multistate tuberculosis pharmacometric model in patients with rifampicin‐treated pulmonary tuberculosis. CPT: Pharmacometrics & Systems Pharmacology, 5, 264–273. 10.1002/psp4.12079 27299939 PMC4873565

[bph15247-bib-0047] Tobin, D. M. , & Ramakrishnan, L. (2008). Comparative pathogenesis of *Mycobacterium marinum* and *Mycobacterium tuberculosis* . Cellular Microbiology, 10, 1027–1039. 10.1111/j.1462-5822.2008.01133.x 18298637

[bph15247-bib-0048] United Nations . (2019). The sustainable development goals report 2019. United Nations Publ. Issued by Dep. Econ. Soc. Aff, DOI: 10.18356/55eb9109-en

[bph15247-bib-0049] US Food and Drug Administration . (2018). Bioanalytical method validation guidance for industry. FDA May

[bph15247-bib-0050] Van der Sar, A. M. , Spaink, H. P. , Zakrzewska, A. , Bitter, W. , & Meijer, A. H. (2009). Specificity of the zebrafish host transcriptome response to acute and chronic mycobacterial infection and the role of innate and adaptive immune components. Molecular Immunology, 46, 2317–2332. 10.1016/j.molimm.2009.03.024 19409617

[bph15247-bib-0051] Van Wijk, R. C. , Ayoun Alsoud, R. , Lennernäs, H. , & Simonsson, U. S. H. (2020). Model‐informed drug discovery and development strategy for the rapid development of anti‐tuberculosis drug combinations. Applied Sciences, 10(7), 2376 10.3390/app10072376

[bph15247-bib-0052] Van Wijk, R. C. , Krekels, E. H. J. , Hankemeier, T. , Spaink, H. P. , & Van der Graaf, P. H. (2016). Systems pharmacology of hepatic metabolism in zebrafish larvae. Drug Discovery Today: Disease Models, 22, 27–34. 10.1016/j.ddmod.2017.04.003

[bph15247-bib-0053] Van Wijk, R. C. , Krekels, E. H. J. , Kantae, V. , Harms, A. C. , Hankemeier, T. , Van der Graaf, P. H. , et al. (2019). Impact of post‐hatching maturation on the pharmacokinetics of exogenous compounds in zebrafish larvae. Scientific Reports, 9, 2149 10.1038/s41598-019-38530-w 30770889 PMC6377609

[bph15247-bib-0054] Van Wijk, R. C. , Krekels, E. H. J. , Kantae, V. , Ordas, A. , Kreling, T. , Harms, A. C. , et al. (2019). Mechanistic and quantitative understanding of pharmacokinetics in zebrafish larvae through nanoscale blood sampling and metabolite modelling of paracetamol. The Journal of Pharmacology and Experimental Therapeutics, 371, 15–24. 10.1124/jpet.119.260299 31371482

[bph15247-bib-0055] Van Wijk, R. C. , Van der Sar, A. , Krekels, E. H. J. , Verboom, T. , Spaink, H. P. , Simonsson, U. S. H. , & Van der Graaf, P . (2020). Quantification of natural growth of two strains of *Mycobacterium marinum* for translational anti‐tuberculosis drug development. Clinical and Translational Science. 10.1111/cts.12793 PMC7719371 32267997

[bph15247-bib-0056] Weerakhun, S. , Hatai, K. , Murase, T. , & Hirae, T. (2008). In vitro and in vivo activities of drugs against *Mycobacterium marinum* in yellowtail *Seriola quinqueradiata* . Fish Pathol., 43, 106–111. 10.3147/jsfp.43.106

[bph15247-bib-0057] Westerfield, M. (2000). The zebrafish book. A guide for the laboratory use of zebrafish (*Danio rerio*). Eugene, OR, USA: University of Oregon Press.

[bph15247-bib-0058] Wicha, S. G. , Clewe, O. , Svensson, R. J. , Gillespie, S. H. , Hu, Y. , Coates, A. R. M. , & Simonsson, U. S. H. (2018). Forecasting clinical dose‐response from preclinical studies in tuberculosis research: Translational predictions with rifampicin. Clinical Pharmacology and Therapeutics, 104, 1208–1218. 10.1002/cpt.1102 29700814 PMC6282494

[bph15247-bib-0059] Wilkins, J. J. , Langdon, G. , McIlleron, H. , Pillai, G. , Smith, P. J. , & Simonsson, U. S. H. (2011). Variability in the population pharmacokinetics of isoniazid in South African tuberculosis patients. British Journal of Clinical Pharmacology, 72, 51–62. 10.1111/j.1365-2125.2011.03940.x 21320152 PMC3141186

[bph15247-bib-0060] World Health Organization . (2012). Tuberculosis laboratory biosafety manual. World Heal. Organ. Publ. ISBN 978 92 41504638 1–60 24404640

