# Supplementary material for: Anti‐tuberculosis effect of isoniazid scales accurately from zebrafish to humans
Source: Br J Pharmacol. 2020 Nov 3;177(24):5518–33. doi: 10.1111/bph.15247 (PMC7707096; doi:10.1111/bph.15247)
Supplement: Supplementary file 1 — Figure S1. Goodness‐of‐fit plots for the pharmacokinetic component of the final pharmacokinetic‐pharmacodynamic model in zebrafish larvae. Figure S2. Model‐based individual prediction of the bacterial burden after isoniazid treatment in zebrafish larvae infected with M. marinum Figure S3. Goodness‐of‐fit plots for the pharmacodynamic component of the final pharmacokinetic‐pharmacodynamic model in zebrafish larvae. Figure S4. Stability of waterborne isoniazid over treatment period. Table S1. Overview of the blood sampling experiment, reporting the number of larvae sampled per timepoint, the total blood volume, and the isoniazid concentration. Model code: Final pharmacokinetic‐pharmacodynamic model in zebrafish infected with M. marinum Model code: Translation to human isoniazid response [file BPH-177-5518-s001.pdf]

Supplementary material to

Anti-tuberculosis effect of isoniazid scales accurately from zebrafish to humans

Rob C. van Wijk[1#], Wanbin Hu[2], Sharka M. Dijkema[1], Dirk-Jan van den Berg[1], Jeremy Liu[1], Rida Bahi[1], Fons J. Verbeek[3], Ulrika S.H. Simonsson[4], Herman P. Spaink[2], Piet H. van der Graaf[1,5], Elke H.J. Krekels[1*]

[1] Division of Systems Biomedicine and Pharmacology, Leiden Academic Centre for Drug Research, Leiden University, Leiden, The Netherlands.

[2] Division of Animal Sciences and Health, Institute of Biology Leiden, Leiden University, Leiden, The Netherlands.

[3] Imaging and Bioinformatics Group, Leiden Institute of Advanced Computer Science, Leiden University, Leiden, The Netherlands.

[4] Department of Pharmaceutical Biosciences, Uppsala University, Uppsala, Sweden.

[5] Certara QSP, Canterbury, UK.

*corresponding author: e.krekels@lacdr.leidenuniv.nl / PO box 9502 2300 RA Leiden The Netherlands / +31 (0)71 527 4505

#current affiliation: Department of Pharmaceutical Biosciences, Uppsala University, Uppsala, Sweden.

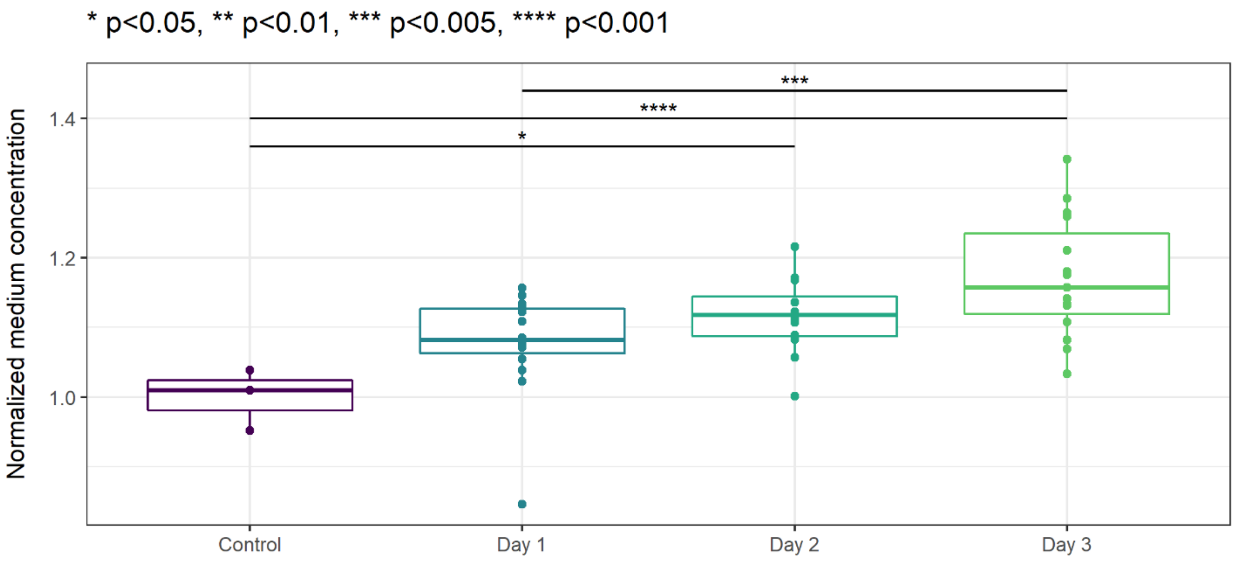

Supplementary figure 1. Stability of waterborne isoniazid over treatment period.

Concentrations were normalized to control. A median increase of 10-15%, probably the result of evaporation of the water in the medium, was assumed to have negligible impact on the absorption rate from the treatment medium, which was therefore kept constant in the pharmacokinetic-pharmacodynamic model. Kruskal-Wallis with Dunn post hoc test: * $p<0.05$, ** $p<0.01$, *** $p<0.005$, **** $p<0.001$

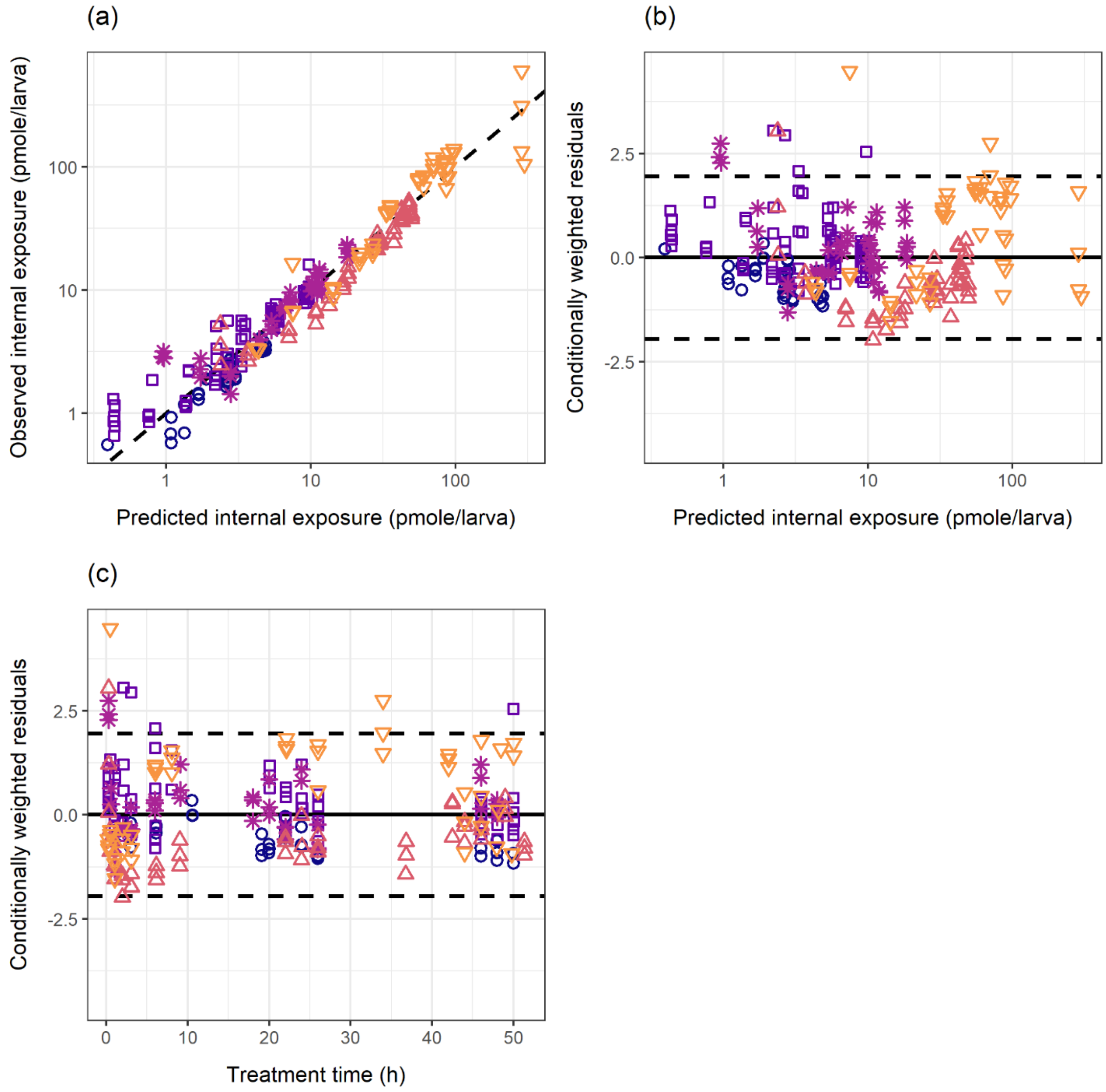

Supplementary figure 2. Goodness-of-fit plots for the pharmacokinetic component of the final pharmacokinetic-pharmacodynamic model in zebrafish larvae. A. Observed vs predicted internal amounts and blood concentrations, dotted line is the line of unity. B. Conditionally weighted residuals vs predicted internal amounts and blood concentrations, solid

line represents zero, dashed lines represent 95% interval between plus and minus 1.96 standard deviation. C. Conditionally weighted residuals vs time, solid line represents zero, dashed lines represent 95% interval between plus and minus 1.96 standard deviation. Symbols represent waterborne isoniazid doses 0.5x (blue circle), 1x (purple square), 2x (lilac star), 5x (orange upward triangle) and 10x MIC (yellow downward triangle). Only small trends for different doses suggest limited bias of predicted internal isoniazid amounts and blood concentrations by the final model.

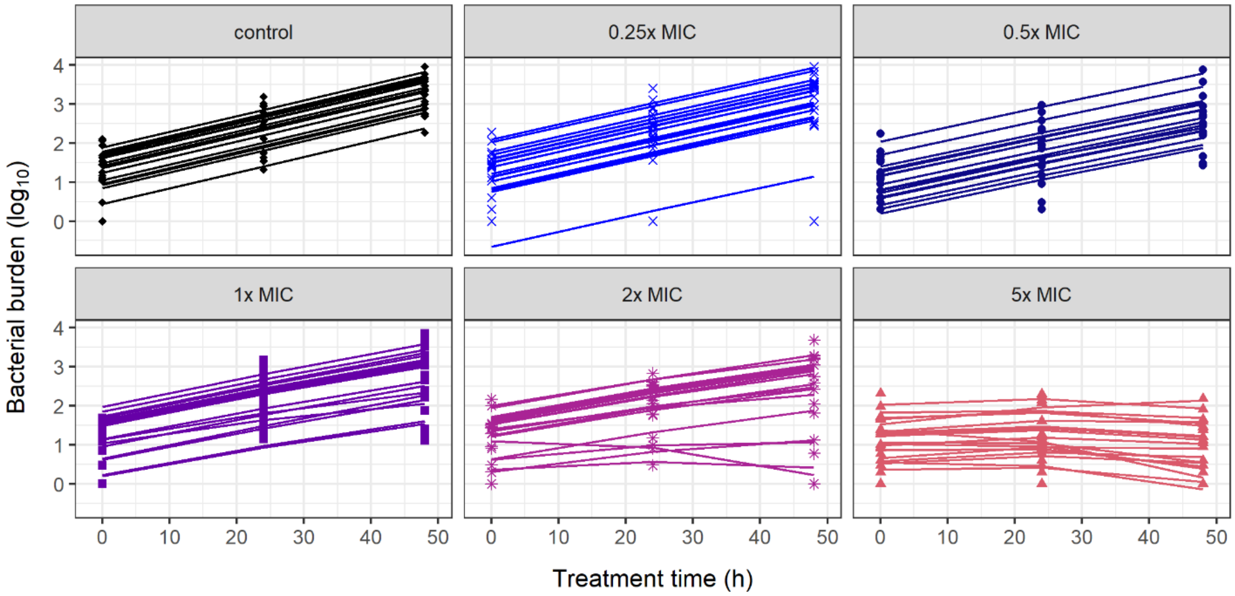

Supplementary figure 3. Model-based individual prediction of the bacterial burden after isoniazid treatment in zebrafish larvae infected with *M. marinum*. The bacterial burden as log_{10}-transformed fluorescent pixel count is shown over treatment time of 50 hours at isoniazid doses in the external treatment medium of 0.25x (light blue crosses and line), 0.5x (blue circles and line), 1x (purple squares and line), 2x (lilac stars and line), and 5x (orange upward triangles and line) MIC, in addition to control (black diamonds and line). Symbols represent observed data, lines connect model predicted bacterial burden for individual zebrafish at each observed time point.

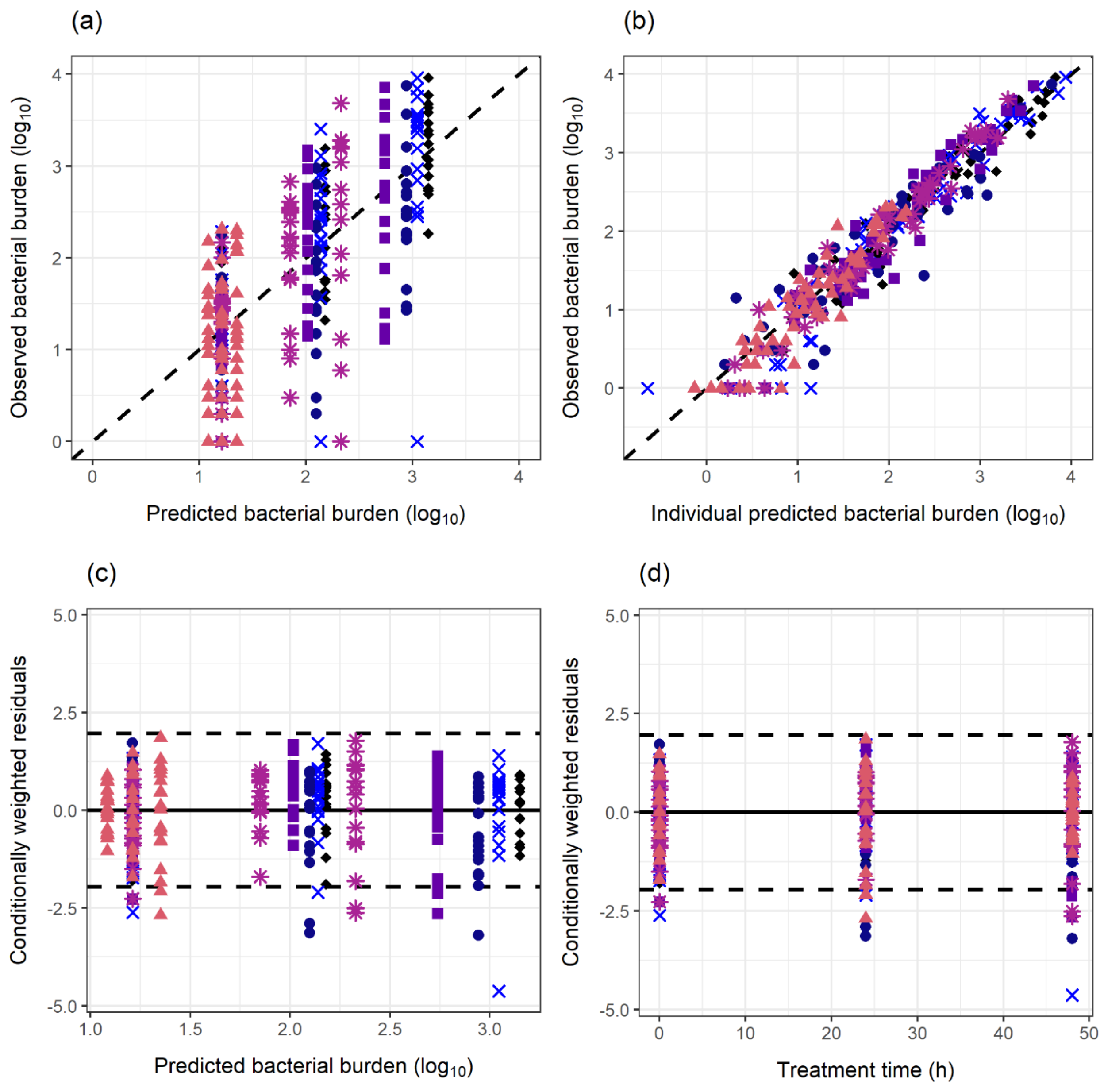

Supplementary figure 4. Goodness-of-fit plots for the pharmacodynamic component of the final pharmacokinetic-pharmacodynamic model in zebrafish larvae. A. Observed vs population predicted bacterial burden, dotted line is the line of unity. B. Observed vs individual predicted bacterial burden, dotted line is the line of unity. C. Conditionally weighted residuals vs predicted bacterial burden, solid line represents zero, dashed lines represent 95% interval between plus and minus 1.96 standard deviation. D. Conditionally weighted residuals vs time,

solid line represents zero, dashed lines represent 95% interval between plus and minus 1.96 standard deviation. Symbols represent doses 0 (black diamond), 0.25 (light blue cross), 0.5x (blue circle), 1x (purple square), 2x (lilac star), and 5x MIC (orange upward triangle). Lack of trends in the goodness-of-fit plots suggest there is no bias in the predictions of bacterial burden by the final model.

Supplementary table 1. Overview of the blood sampling experiment, reporting the number of larvae sampled per timepoint, the total blood volume, and the isoniazid concentration.

**Mean sampling time (hh:mm)**	**Number of larvae sampled**	**Total blood volume (nL)**	**Blood concentration (pmole·μL^{-1} or µM)**
47:57	31	15.25	133.34
48:08	19	7.058	308.84
48:26	27	11.38	602.66
49:47	24	10.23	105.13

Model code: Final pharmacokinetic-pharmacodynamic model in zebrafish infected with *M. marinum*

```
$PROBLEM PKPD
$INPUT ID TIME AMT DV EVID MDV CMT BQL AQL DOSE AGE AGE_H XEXP
BLOOD FLAG
$DATA RW04_M07_PKPD_dataset_v1.csv IGNORE=@ IGNORE=(BQL.EQ.1)
IGNORE=(AQL.EQ.1)

; time in hours of exposure
; amt in uM (concentration)
; DV in pmole / larvae or pmole / uL (uM) / log10(bac burden)
; Vd in uL
; AGE_H age in hpf

$SUBROUTINE ADVAN13 TOL=9

$MODEL
COMP ;CMT 1 dosing
COMP ;CMT 2 isoniazid in larva
COMP ;CMT 3 bacterial burden measured in log10(fluorescence pixels)

$PK

;-----PK-----

TVKE = THETA(1)           ;first order elimination rate
TVV2 = THETA(2)            ;distribution volume in uL/larva
TVKA = THETA(3) / 1e2    ;absorption at start of experiment
KA_GI = THETA(4)           ;effect of GI opening between 3 and 4 dpf on absorption
KA_HPF = THETA(5)         ;effect of age in hours post fertilization on absorption

;age effect on KA
KA = TVKA * KA_HPF**(AGE_H / 101)   ;median age is 101 hpf

;GI effect on KA
IF(AGE_H.LE.90) KIN = KA * DOSE
IF(AGE_H.GT.90) KIN = KA * DOSE * (1 + KA_GI)

KE = TVKE
V2 = TVV2

;-----PD-----

KG = THETA(6)                                   ;exponential growth rate
```

```
INOC = THETA(7) * 1e1                          ;inoculum estimated
SLP = (THETA(8) / 1e2) * EXP(ETA(2))     ;slope (linear drug effect)

A_0(3) = INOC * EXP(ETA(1))                  ;inoculum set as compartment initial value

$DES
DADT(1) = 0                           ;dosing compartment
DADT(2) = KIN - KE * A(2)    ;larva compartment isoniazid

;drug effect

;Distribution volume is estimated based on 5 dpf blood samples, but is expected to be lower with
younger larvae of 3 and 4 dpf.
;It is here assumed that the distribution volume scales with total volume.

IF(AGE.EQ.3) THEN
V = V2 * 253/300         ;taking total volume into account (DOI: 10.1364/BOE.8.002611)
ENDIF
IF(AGE.EQ.4) THEN
V = V2 * 263/300         ;taking total volume into account (DOI: 10.1364/BOE.8.002611)
ENDIF
IF(AGE.EQ.5) THEN
V = V2                          ;taking total volume into account (DOI: 10.1364/BOE.8.002611)
ENDIF

C = A(2)/V

EFF = 0 ;for C is zero or NA
IF(C.GT.0) THEN
EFF = SLP * C
ENDIF

DADT(3) = KG * A(3) * (1 - EFF) ;bacterial burden (log10)

$ERROR

;-----PK-----

IF(FLAG.EQ.0.AND.BLOOD.EQ.0) THEN
IPRED = A(2)
Y = IPRED * (1 + EPS(1)) + EPS(2) ; comb error
W = SQRT(IPRED**2*SIGMA(1,1)**2    + SIGMA(2,2)**2)
IRES = DV - IPRED
ENDIF

IF(FLAG.EQ.0.AND.BLOOD.EQ.1) THEN
```

```
IPRED = A(2) / V2
Y = IPRED * (1 + EPS(3)) + EPS(4) ; comb error
W = SQRT(IPRED**2*SIGMA(3,3)**2    + SIGMA(4,4)**2)
IRES = DV - IPRED
ENDIF

;-----PD-----

IF(FLAG.EQ.1) THEN
IPRED = LOG10(A(3)+0.00001)
Y = IPRED + W
W = EPS(5)
IRES = DV - IPRED
ENDIF

IF(W.EQ.0)W=1
IWRES = IRES/W

$THETA
(0, 0.58)      ; 1 KE
(0, 0.325)     ; 2 V2
(0, 0.355)     ; 3 KA
(0, 0.178)     ; 4 KA_GI
(1, 7.45)      ; 5 KA_HPF
(0, 0.093)     ; 6 KG
(0, 1.6)       ; 7 *1e1 INOC
(0, 0.991)     ; 8 /1e2 SLP

$OMEGA 1.64     ;IIV INOC
$OMEGA 0.227    ;IIV SLP

$SIGMA 0.0611   ; 1 prop homogenate INH
$SIGMA 0.59     ; 2 add homogenate INH
$SIGMA 0.485    ; 3 prop blood INH
$SIGMA 0 FIX    ; 4 add blood INH
$SIGMA 0.125    ; 5 prop bacterial burden

$ESTIMATION METHOD=1 INTER MAXEVAL=8000 NOABORT PRINT=10 NSIG=3
SIGL=9 POSTHOC
$COVARIANCE PRINT=E

$TABLE ID TIME KIN KA TVKA KE V2 KA_GI KA_HPF KG INOC SLP C V ETA(1)
ETA(2) DV EVID MDV CMT BQL AQL DOSE AGE AGE_H BLOOD XEXP FLAG IPRED
IRES IWRES CWRES NOPRINT ONEHEADER FILE=tab1
```

Model code: Translation to human isoniazid response

```
$PROBLEM PK INH

$INPUT ID TIME AMT DV EVID MDV CMT WT METAB DOSE
$DATA 20190905_RW04_M08_SIM_dataset_PKPD_v4.csv IGNORE=@

; time in hours
; amt in mg
; dv in mg/L (--> transform linear slope from zebrafish in uM)
; subjects are all male and HIV negative (no covariates)
; METAB is 1 for fast and 0 for slow metabolizers

$SUBROUTINE ADVAN13 TOL=9

$MODEL

COMP(ABS DEFDOSE)
COMP(CENTRAL DEFOBS)
COMP(PERIPH)
COMP(BAC)

$PK

;PK (simulated from Wilkins et al, Br. J. Clin. Pharmacol. 72, 51–62 (2011))

;typical values
TVKA = THETA(1)          ;typical value absorption rate constant
TVALAG2 = THETA(2)   ;typical value absorption lag time to compartment 2

TVV2 = THETA(3)          ;typical value distribution volume central compartment
TVV3 = THETA(4)          ;typical value distribution volume peripheral compartment
TVQ = THETA(5)            ;typical value intercompartmental clearance

TVCLS = THETA(6)       ;typical value clearance slow metabolizers
TVCLF = THETA(7)       ;typical value clearance fast metabolizers

;PK parameters

KA = TVKA
ALAG2 = TVALAG2 * EXP(ETA(1))
V2 = TVV2 * (WT/70)**1.00 * EXP(ETA(2))
V3 = TVV3 * (WT/70)**1.00
Q = TVQ * EXP(ETA(3))
CLS = TVCLS * (WT/70)**0.75 * EXP(ETA(4))
CLF = TVCLF * (WT/70)**0.75 * EXP(ETA(4))
```

```
;microconstants

KEF = CLF / V2
KES = CLS / V2
KE = KES * (1 - METAB) + KEF * (METAB)   ;distinguish between fast and slow metabolizer
K23 = Q / V2
K32 = Q / V3

S2 = V2

;PD (from PKPD model of isoniazid in zebrafish)

;estimated exponential growth (kg = 0.093007) is used to back calculate -48h from inoculum
(inoc = 16.276) which is moment of infection in zebrafish with 200 CFU M marinum
;and subsequently to CFU/mL

FL_start = 16.276 * exp(-48 * 0.093007)
CFU_start = 200
f_FL_CFU = CFU_start/FL_start          ;factor to convert fluorescence to CFU
CFU_INOC = 16.276 * f_FL_CFU
f_INOC_CFU = 4888538/CFU_INOC   ;mean inoculum from digitized papers

TVKG = THETA(8)      ;exponential growth rate
TVINOC = THETA(9)  ;inoculum estimated
TVSLP = THETA(10)   ;slope (linear drug effect)

KG = TVKG
INOC = TVINOC * EXP(ETA(5))
SLP0 = TVSLP * EXP(ETA(6))

A_0(4) = INOC * f_FL_CFU * f_INOC_CFU  ;inoculum in CFU/mL

$DES

DADT(1) = -KA * A(1)
DADT(2) = KA * A(1) - KE * A(2) - K23 * A(2) + K32 * A(3)
DADT(3) = K23 * A(2) - K32 * A(3)

C = A(2)/V2

;translational factors (Wicha et al, Clin. Pharmacol. Ther. 104:6, 1208-1218 (2018))

;1. MIC
```

```
MIC_fac = 0.2/15        ;correct for MIC of INH for MTB (0.2 mg/L, breakpoint MIC from Schön et al, J. Antimicrob. Chemother. 64, 786–793 (2009)) in comparison to MM (15 mg/L) by increasing sensitivity

;2. Stage of infection
INFEC_fac = 22.2/8.55  ;correct for difference in effect on logarithmic (zebrafish infection, mostly F) and stationary (clinical infection, mostly S and N) by taking ratio of maximal kill rate of isoniazid on F and S state (no isoniazid quantified on N state, Clewe et al, J. Antimicrob. Chemother. 73, 437–447 (2018))

SLP = SLP0 / MIC_fac / INFEC_fac
EFF = SLP * C

DADT(4) = KG * A(4) * (1 - EFF)

$ERROR

IF(CMT.NE.4) THEN
IPRED = LOG(F + 0.0001)
Y = IPRED + EPS(1)
ENDIF

IF(CMT.EQ.4) THEN
IPRED = LOG10(A(4) + 0.0001)
Y = IPRED + EPS(2)
ENDIF

$THETA
1.85            ;1 KA h-1
0.180           ;2 ALAG2 h
57.7            ;3 V2 L
1730            ;4 V3 L
3.34            ;5 Q L/h
9.70            ;6 CLS L/h slow metabolizer
21.6            ;7 CLF L/h fast metabolizer
0.093007        ;8 KG
16.276          ;9 INOC
0.07227485  ;10 SLP 0.0099117/0.137139 (convert slope from uM to mg/L by dividing by mol. weight)

$OMEGA
0.781456  ;IIV ALAG2 reported %CV 88.4
0.027225  ;IIV V2 reported %CV 16.5
0.866761  ;IIV Q reported %CV 93.1
0.033856  ;IIV CL reported %CV 18.4
1.64          ;IIV INOC
```

```
0.227      ;IIV SLP

$SIGMA
0.042025  ;additive on natural log scale, sd = 0.205 mg/L
0.124      ;additive on natural log scale

$SIMULATION (12345) ONLYSIM SUBPROBLEM=1

$TABLE ID TIME AMT DV EVID MDV CMT WT METAB DOSE
TVKA TVALAG2 TVV2 TVV3 TVQ TVCLS TVCLF TVKG TVINOC TVSLP
KA ALAG2 V2 V3 Q CLS CLF KG INOC SLP SLP0 f_FL_CFU ETA1 ETA2 ETA3 ETA4
ETA5 ETA6
IPRED NOPRINT ONEHEADER FILE=tab21
```
